# *Pseudomonas syringae* addresses distinct environmental challenges during plant infection through the coordinated deployment of polysaccharides

**DOI:** 10.1093/jxb/erab550

**Published:** 2021-12-14

**Authors:** Pilla Sankara Krishna, Stuart Daniel Woodcock, Sebastian Pfeilmeier, Stephen Bornemann, Cyril Zipfel, Jacob George Malone

**Affiliations:** 1 Department of Molecular Microbiology, John Innes Centre, Norwich Research Park, Norwich NR4 7UH, UK; 2 The Sainsbury Laboratory, University of East Anglia, Norwich Research Park, Norwich NR4 7UH, UK; 3 Department of Biological Chemistry, John Innes Centre, Norwich Research Park, Norwich NR4 7UH, UK; 4 University of East Anglia, Norwich Research Park, Norwich NR4 7TJ, UK; 5 Lancaster University, UK

**Keywords:** Biofilm, cyclic-di-GMP, exopolysaccharides, lipopolysaccharide, plant infection *Pseudomonas syringae*, surface adhesion

## Abstract

Prior to infection, phytopathogenic bacteria face a challenging environment on the plant surface, where they are exposed to nutrient starvation and abiotic stresses. Pathways enabling surface adhesion, stress tolerance, and epiphytic survival are important for successful plant pathogenesis. Understanding the roles and regulation of these pathways is therefore crucial to fully understand bacterial plant infections. The phytopathogen *Pseudomonas syringae* pv. *tomato* (*Pst*) encodes multiple polysaccharides that are implicated in biofilm formation, stress survival, and virulence in other microbes. To examine how these polysaccharides impact *Pst* epiphytic survival and pathogenesis, we analysed mutants in multiple polysaccharide loci to determine their intersecting contributions to epiphytic survival and infection. In parallel, we used qRT–PCR to analyse the regulation of each pathway. *Pst* polysaccharides are tightly coordinated by multiple environmental signals. Nutrient availability, temperature, and surface association strongly affect the expression of different polysaccharides under the control of the signalling protein genes *ladS* and *cbrB* and the second messenger cyclic-di-GMP. Furthermore, functionally redundant, combinatorial phenotypes were observed for several polysaccharides. Exopolysaccharides play a role in mediating leaf adhesion, while α-glucan and alginate together confer desiccation tolerance. Our results suggest that polysaccharides play important roles in overcoming environmental challenges to *Pst* during plant infection.

## Teaser

*Pseudomonas syringae*uses the coordinated deployment of polysaccharides to address environmental challenges during plant colonization. Functional redundancy renders individual polysaccharides dispensable during plant infection, but their combined loss impedes pathogenicity.

## Introduction

*Pseudomonas syringae* is a Gram-negative, plant pathogenic bacterium and a widely used model system to understand plant–microbe interactions (Hirano and Upper, 2000; Xin *et al.*, 2018). Taxonomic studies indicate that *P. syringae* is in fact a diverse phylogenetic group containing >15 species and >60 pathovars (Young, 2010). Pathovars of *P. syringae* infect almost all economically important crop plants and are considered to be one of the most common and damaging bacterial plant pathogens that infect the phyllosphere (Xin *et al.*, 2018). *Pseudomonas syringae* pathogenesis is attributed to a repertoire of virulence factors, including secretion systems, phytotoxins and phytohormones, quorum-sensing pathways, ice nucleation agents, cell wall-degrading enzymes, and exopolysaccharides (EPSs) (Hirano and Upper, 2000; Pfeilmeier *et al.*, 2016a; Xin *et al.*, 2018). Plant infection by *P. syringae* consists of epiphytic and endophytic phases (Xin and He, 2013). Survival on plant surfaces such as leaves, stems, or fruits is referred as the epiphytic phase, while the endophytic phase describes bacterial entry into the plant tissue and colonization of the intercellular apoplastic space. Bacteria face a challenging environment on the plant surface, where they are routinely exposed to mechanical, temperature, and desiccation stresses, nutrient starvation, and UV irradiation (Andrews and Harris, 2000; Wu *et al.*, 2012; Djonovic *et al.*, 2013; Pfeilmeier *et al.*, 2016a). Thus, adhesion to plant surfaces, stress tolerance, and epiphytic survival are likely to be important for successful pathogenesis.

Extracellular matrix and cell envelope components such as lipopolysaccharide (LPS) and EPS molecules interact directly with the host/plant surface and hence are vital in establishing an interaction during the epiphytic phase (Mhedbi-Hajri *et al.*, 2011; King and Roberts, 2016). Genetic studies have shown that LPS production is required for efficient host colonization and for full virulence in plant pathogenic *Pseudomonas* spp., *Erwinia amylovora*, and *Burkholderia cenocepacia* (Berry *et al.*, 2009; Khodai-Kalaki *et al.*, 2015; Kutschera *et al.*, 2019). Similarly, EPS production has been implicated in epiphytic survival of *P. syringae* and *Xanthomonas* spp., and in the wider plant microbiome (Yu *et al.*, 1999; Dunger *et al.*, 2007). It has also been suggested to contribute to bacterial freeze–thaw resistance (Wu *et al.*, 2012). The widely studied model plant pathogen *P. syringae* pv. *tomato* str. DC3000 (*Pst*) contains multiple polysaccharide gene clusters that can potentially contribute to biofilm matrix formation, including alginate, Wss cellulose, Psl, and α-glucan (Winsor *et al.*, 2016).

Alginate is a copolymer of acetylated β-1,4-linked d-mannuronic acid and l-glucuronic acid that contributes to antibiotic and immune system protection in the human pathogen *Pseudomonas aeruginosa* (Hentzer *et al.*, 2001; Pier *et al.*, 2001). Alginate is not considered essential to *Pseudomonas* biofilm formation (Wozniak *et al.*, 2003; Laue *et al.*, 2006), but has been implicated in epiphytic fitness and virulence in *P. syringae* pathovars *actinidiae* and *syringae* (Yu *et al.*, 1999; McAtee *et al.*, 2018; Helmann *et al.*, 2019). Cellulose is a homopolymer made of β-d-glucose monomers and is a primary component of the biofilm matrix of *Pst* and many other bacteria (Serra *et al.*, 2013; Prada-Ramirez *et al.*, 2016; Farias *et al.*, 2019). Cellulose has been suggested to play an important role in the transition between epiphytic and pathogenic phases of leaf association (Arrebola *et al.*, 2015). Similarly, acetylated cellulose (Wss) contributes substantially to root association of the rhizosphere bacterium *P. fluorescens* (Gal *et al.*, 2003). Psl is a pentasaccharide polymer of d-mannose, d-glucose, and l-rhamnose subunits that plays essential roles in biofilm formation, adhesion, motility, and stress protection in *P. aeruginosa* (Byrd *et al.*, 2009; Billings *et al.*, 2013; Periasamy *et al.*, 2015). The function of Psl in *P. syringae* remains relatively unclear, although it has recently been implicated in swarming motility and pathogenicity regulation in the *P. syringae* mango pathovar UMAF0158 (Heredia-Ponce *et al.*, 2020).

GlgE-derived α-glucan is a glycogen-like homopolymer of glucose monomers with α-1,4 glycosidic links and α-1,6-linked branch points (Edstrom, 1972). α-Glucan is a ubiquitous carbon store and plays important roles in desiccation stress tolerance in *P. aeruginosa* (Woodcock *et al.*, 2021), and virulence in *Mycobacterium tuberculosis* (Sambou *et al.*, 2008; Koliwer-Brandl *et al.*, 2016). Recently, α-glucan has been implicated as an EPS in the virulent kiwi pathovar *P. actinidiae* (Ghods *et al.*, 2015). The ubiquitous compatible solute trehalose is not only a precursor of α-glucan (Woodcock *et al.*, 2021) but also contributes to osmotic stress tolerance during epiphytic survival by *P. syringae* (Freeman *et al.*, 2010). Finally, levan is a β-2,6 polyfructan with extensive branching through β-2,1 linkages (Li and Ullrich, 2001; Laue *et al.*, 2006). Levan has not been implicated in biofilm formation or epiphytic survival. Instead, it has been suggested to function as a storage molecule that may be produced in the apoplast (Laue *et al.*, 2006; Yu *et al.*, 2013).

LPS molecules are complex glycoconjugate molecules whose biosynthesis, packing, and role in pathogenesis and immune evasion is well understood in animal/human pathosystems but less explored in plant pathogens. The *Pst* genome encodes at least four LPS biosynthesis operons, including genes for WaaP and Waa proteins alongside *wapQ/wapG* and *wbpL* as per annotations in the *Pseudomonas* genome database pseudomonas.com (Winsor *et al.*, 2016). In *Erwinia amylovora*, LPS production contributes to virulence and oxidative stress protection (Berry *et al.*, 2009). Similarly, in *Burkholderia cenocepacia* and *Xylella fastidiosa*, virulence is compromised in LPS mutants (Khodai-Kalaki *et al.*, 2015; Rapicavoli *et al.*, 2018). Recent studies have shown that *Pst* cells lacking a *wbpL* orthologue failed to produce *O*-polysaccharide and exhibited reduced apoplast colonization and pathogenesis (Kutschera *et al.*, 2019). The LPS core kinase gene *waaP* can be deleted in several Gram-negative bacteria such as *Escherichia coli* and *Salmonella enterica* but not in *P. aeruginosa* (DeLucia *et al.*, 2011). Finally, PSPTO4998 of *Pst* encodes a putative WaaP family LPS kinase (*wapQ/wapG/inaA*) but is poorly characterized in *P. syringae*.

*Pst* phytotoxins and effector proteins and their roles in immune suppression have been extensively studied. However, to obtain a comprehensive understanding of plant infection, we also need to unravel the relationship between bacterial survival, stress tolerance, and pathogenesis. In this study, we build on prior research on cellulose and alginate production in *Pst* (Keith *et al.*, 2003; Farias *et al.*, 2019; Perez-Mendoza *et al.*, 2019) to examine the intersecting roles of multiple *Pst* polysaccharide molecules in mediating bacterial infection and enabling epiphytic survival. We hypothesize that bacterial polysaccharides are differentially expressed in response to the environmental cues and play important roles in abiotic stress tolerance and epiphytic survival during plant infection. To test this, *Pst* mutants were constructed in the alginate (*alg*), *psl*, cellulose (*wss*), and α-glucan (*glg/tre*) pathways, and the putative LPS kinase gene *wapQ*, and their relative contributions to phenotypes including colony morphology, abiotic stress tolerance, epiphytic survival, and plant infection were defined.

The polysaccharide pathways in *Pst* are each tightly regulated by external environmental cues. In particular, *alg* and *psl* expression was shown to be strongly nutrient dependent, while *wss* production was activated by low temperatures and overproduction of the second messenger molecule bis-(3ʹ-5ʹ)-cyclic dimeric GMP (or cyclic-di-GMP) (Jenal *et al.*, 2017). Expression of the *glg* trehalose/α-glucan locus is induced by surface association, under the control of the global regulators *ladS* and *cbrB* (Sonnleitner *et al.*, 2009; Grenga *et al.*, 2017). Strikingly, we observed a substantial degree of functional redundancy and phenotypic interaction between the polysaccharide molecules in our study. While disruption of individual polysaccharide loci had little effect on *Pst* plant infection, mutation of multiple loci led to compromised infection or delayed disease onset for several different combinations. Similarly, we observed combinatorial phenotypic effects for several loci, suggestive of functional redundancy. Alginate and α-glucan combine to confer desiccation stress tolerance, while disruption of EPS genes only impacted leaf surface adhesion when combined with a *wapQ* deletion. Our data suggest that bacterial polysaccharides play important, intersecting roles in enabling infection by plant pathogens.

## Materials and methods

### Strains and growth conditions

Unless otherwise stated, all strains were grown at 28 °C with shaking. Bacterial strains and plasmids used in this study are listed in Table 1. King’s B (KB) medium (King *et al.*, 1954), Lennox (L) medium (Sambrook and Russel, 2001), soya flour mannitol medium (SFM) [2% soya flour (SF) and 2% mannitol], and M9 medium [M9 salts (Sambrook and Russel, 2001) supplemented with 0.4% glucose, 0.4% casamino acids, and 50 µM FeCl_3_] were used for culturing and for *in vitro* assays. Antibiotics were used at a final concentration of gentamicin at 25 μg ml^–1^, tetracycline at 12.5 μg ml^–1^, and kanamycin at 25 μg ml^–1^ for selection of mutants or during genetic manipulations. Final concentrations of X-gal of 40 μg ml^–1^ and isopropyl-β-d-thiogalactopyranoside (IPTG) of 1 mM were used for blue–white screening the of reporter strain.

**Table 1.** List of strains used in this study

*P. syringae* pv *tomato* DC3000 (*Pst*)		
Strain name	Description	Reference
WT	Rifampicin resistant *Pst* considered as wild type	Buell *et al.* (2003)
∆*alg*	Strain with *alg*G (*PSPTO1238*) and *alg*X (*PSPTO1237*) deletion	Lammertz *et al.* (2019)
∆*psl*	Strain with *pslD* (*PSPTO3531*) and *pslE* (*PSPTO3532*) deletion	Lammertz *et al.* (2019)
∆*wss*	Strain with *wssB* (*PSPTO1027*) and *wssC* (PSPTO1028) deletion	Lammertz *et al.* (2019)
∆*treS*	Strain with *glgE* (*PSPTO2760*), *treS* (*PSPTO2761*), and *glgB* (*PSPTO2762*) deletion	This study
∆*treY/Z*	Strain with *glgA* (*PSPTO3125*), *treZ* (*PSPTO3126*), *malQ* (*PSPTO3127*), *treY* (*PSPTO3128*) and (*PSPTO3129*), and *glgX* (*PSPTO3130*) deletion	This study
∆*treS* ∆*treY/Z*	A combination of ∆*treS* and ∆*treY/Z*	This study
∆*alg ∆psl*	Strain with *alg*G-*X* (*PSPTO1238, PSPTO1237*) and *pslD-E* (*PSPTO3531, PSPTO3532*) deletion	Lammertz *et al.* (2019)
*∆alg ∆wss*	Strain with *alg*G (PSPTO1238) and *alg*X (*PSPTO1237*) and *wssB-C* (*PSPTO1027, PSPTO1028*) deletion	Lammertz *et al.* (2019)
*∆psl ∆wss*	Strain with *pslD-E* (*PSPTO3531, PSPTO3532*) and *wssB-C* (*PSPTO1027, PSPTO1028*) deletion	Lammertz *et al.* (2019)
*∆alg ∆treS* ∆*treY/Z*	Strain with *alg*G-X (*PSPTO1238*, *PSPTO1237*) deletion combined with ∆*treS* ∆*treY/Z*	Lammertz *et al.* (2019)
∆*EPS* (*∆alg ∆psl ∆wss*)	Strain with *alg*G-X, *pslD-E*, and *wssB-C* deletion	Lammertz *et al.* (2019)
*∆wapQ* (*∆PSPTO4998*)	Strain with deletion of an orthologue of *wapQ (*∆*PSPTO4998*)	This study
∆*EPS*+∆*wapQ*	A combination of ∆*EPS* and ∆*wapQ*	This study
*Pst* P*glgA-lacZ*	Reporter strain developed by genome integration of the construct into neutral site using mini Tn*7*. Reporter construct was generated by cloning *lacZ* gene under the control of *glgA* promoter.	This study
Tn::*cbrB*	*Pst* P*glgA-lacZ* strain with identified transposon insertion in *cbrB* gene	This study
Tn::*ladS*	*Pst* P*glgA-lacZ* strain with identified transposon insertion in *ladS* gene	This study
Tn::*PSPTO1866*	*Pst* P*glgA-lacZ* strain with identified transposon insertion in *PSPTO1866* gene	This study
Plasmids		
pTS1	Tet^R^, suicide vector; *ColE1*-replicon, *IncP-1*, *Mob*, *lacZ*	Scott *et al.* (2017)
pBBR-*wspR19*	Kan^R^, resistant and di-guanylate cyclase (*wspR19*)-expressing plasmid	Pfeilmeier *et al.* (2016b)

### Mutagenesis and genetic manipulation

Gene deletion vectors were constructed by amplifying the upstream and downstream regions flanking the desired gene from the *Pst* genome using primers nos 1–72, listed in Supplementary Table S1. Amplified up- and downstream flanking regions were then cloned into the multiple cloning site of the suicide vector pTS1 (Scott *et al.*, 2017). *Pst* cells were transformed using electroporation with the appropriate deletion vectors following the method described in Choi *et al.* (2006). Single crossover integrations of each plasmid into the chromosome were selected on tetracycline plates and re-streaked before single colonies were grown overnight in KB medium without selection. Double crossovers were counterselected by plating serial dilutions onto L agar containing 10% (w/v) sucrose. Deletion mutants were confirmed by PCR using corresponding primers labelled test-F and test-R in Supplementary Table S1. To produce the *Pst*-P*glgA-lacZ* reporter, the promoter region of *Pst glgA* (*PSPTO3125*) including several codons of the ORF was amplified using primers nos 73 and 74, given in Supplementary Table S1 and cloned between the *Bam*HI and *Hin*dIII sites of pUC18-mini-*Tn7*T-Gm-LacZ10 (Choi and Schweizer, 2006).

### Transposon mutagenesis screening

The plasmid pALMAR3 was introduced into *Pst*-P*glgA-lacZ* via biparental mating with *E. coli* S17-1. Mariner transposon insertion mutants were selected by plating onto L agar containing gentamycin, tetracycline, and X-Gal. Colonies showing changes in LacZ activity were re-streaked and the location of the transposon in each case was determined by arbitrary PCR (O’Toole and Kolter, 1998) using primers 75–78 given in Supplementary Table S1.

### Extraction and NMR analysis of metabolites

Overnight *Pst* cultures were grown in M9 medium, then adjusted to a cell density of 0.5 (OD_600_) in phosphate-buffered saline (PBS). Mixed cellulose ester filter discs (Merck Millipore) were placed on the surface of M9 agar plates and coated with the diluted cell suspensions. Plates were then incubated at 28 °C for 48 h before cells were harvested from each disc and resuspended in 5 ml of double-distilled water (ddH_2_O) in a 15 ml plastic tube by vigorous vortexing. Vacuum dried cells were weighed (DW), resuspended in ddH_2_O, and boiled at 95 °C for 20 min. Boiled cells were then centrifuged (10 000 *g*, 10 min) and the resulting supernatant was subject to additional boiling and centrifugation steps to remove any remaining insoluble contaminants. The supernatant, now containing the total water-soluble metabolite content of the cell sample, was dried under vacuum for 16 h. The resulting dried pellet was resuspended in 1200 μl of D_2_O and analysed by ^1^H-NMR spectroscopy (Bruker AVANCE III 400 spectrometer), at room temperature with water suppression. Metabolite chemical shifts were recorded as parts per million (ppm) relative to 0.5 mM trimethylsilyl propanoic acid (TMSP; 0.00 ppm). Spectra were analysed using Topspin 3.0 (Bruker), and the concentrations of metabolites were established through the manual integration of peaks relative to TMSP. The resonances were assigned based on previously established spectra (Usui *et al.*, 1974; Woodcock *et al.*, 2021)

### Plant infection assays and bacterial load estimation

*Arabidopsis thaliana* Col-0 (Col-0) plants were grown in a controlled-environment room in short-day conditions: 10 h light, 22 °C, 70% relative humidity. Cell cultures were grown overnight in L medium, then resuspended in 10 mM MgCl_2_ and adjusted to a final density (OD_600_) of 0.2, equivalent to 1 × 10^8^ colony-forming units (CFU) ml^–1^. Then 0.04% (w/v) of Silwet® L-77 (phytotech labs) was added to the cell suspension as a surfactant just before spraying on the plants. Four- to five-week-old plants were infected using a hand-held sprayer until all leaves appeared wet. For leaf infiltration experiments, cells at a final OD_600_ of 0.02 (equivalent to 10^7^ cells ml^–1^) were gently infiltrated into the leaf apoplast using a 1 ml syringe until leaves appeared wet. For calculating bacterial load, two 7 mm diameter leaf discs (area 0.384 cm^2^) were collected for each sample and decanted into 200 µl of sterile 10 mM MgCl_2_. Post-infiltration/spray infection, bacterial suspensions were allowed to absorb/dry onto leaf surfaces for ~1 h before initial (0 D) samples were collected. Subsequent samples were collected after 24 h (1 D), 48 h (2 D), etc., up to 5 d over the course of each infection. Samples were lysed using a Geno/Grinder 2010 high-throughput tissue homogenizer, with two 3 mm glass beads and two cycles of 1700 vibrations min^–1^ with a 1 min break. Bacterial counts were determined by plating 10-fold serial dilutions on L agar plates with 50 µg ml^–1^ rifampicin and 20 µg ml^–1^ nystatin. A minimum of eight plants were used for each condition, and assays were run at least twice independently. Bacterial load was calculated and presented as CFU per unit leaf area (CFU cm^–2^).

### Growth assays

Bacterial growth was measured in a microplate spectrometer using a minimum of five biological replicates and is presented as the mean ±SD. A 150 μl aliquot of the indicated growth medium in each case was added to the wells of clear-bottomed, black-walled 96-well microplates. Growth was initiated by the addition of 5 μl of overnight cell culture (L medium), to obtain a starting OD_600_ of 0.01. Plates were incubated statically at 28 °C, and agitated (500 rpm, 5 s) prior to each data acquisition step. The OD was measured at 600 nm. Experiments were conducted at least twice independently.

### Osmotic stress assays

Overnight *Pst* cultures were grown in M9 medium, then diluted to an OD_600_ of 0.01 in M9 medium. A 5 µl aliquot of each diluted sample was used to inoculate 150 µl of M9 medium. To examine osmotic stress conditions, growth media were supplemented with 0.35 M NaCl. Growth was monitored by measuring the OD at 600 nm every hour for 6 d. Assays were conducted in triplicate and repeated at least twice independently, with a representative sample shown in each case.

### Desiccation tolerance assays

Desiccation tolerance assays were conducted following the method as previously described (Woodcock *et al.*, 2021). Overnight *Pst* cultures were grown in M9 medium, then diluted to an OD_600_ of 0.1 in PBS. A 10 µl aliquot of each culture was spotted onto 15 mm grade 1 Whatman filter discs. After drying for 1 min at room temperature, discs were placed onto M9 agar plates and incubated at 28 °C for 4 h to enable bacteria to recover and begin dividing. After incubation, the filter discs were subjected to controlled desiccation in tightly sealed bell chambers containing either water (100% relative humidity) or a saturated solution of NaCl (75% relative humidity) (de Goffau *et al.*, 2009) for 2 h. Bacteria were then recovered from filter discs in 3 ml of PBS and serially diluted before spreading onto L agar plates. CFU were determined for each strain, then log_10_(CFU) values were analysed by linear mixed modelling using restricted maximum likelihood (REML) following the methodology described in Woodcock *et al.* (2021). Assays were conducted in triplicate and repeated at least twice independently.

### Assessment of colony morphology

To assess the colony morphology of the *Pst* mutant strains, overnight cultures were grown in L medium, resuspended in 10 mM MgCl_2_ solution, and final densities were adjusted to an OD_600_ of 0.1. Agar plates were prepared and allowed to dry for 45 min in a sterile flow chamber. KB and SFM (Kieser *et al.*, 2000) plates were prepared, with Congo red (CR) dye added as appropriate (30 μg ml^–1^). A 5 μl aliquot of each culture was spotted onto the agar surface and allowed to dry in a sterile flow chamber. Plates were then incubated at 28 °C for the indicated period before photographing, with a representative image shown in each case. For temperature treatments, plates were incubated at 28 °C for 24 h to enable colony establishment before plates were transferred to the designated temperature for a further incubation period as stated in the text.

### Leaf surface adhesion and surface survival assays

Adhesion assays were performed according to Arrebola *et al.* (2015) with minor modifications. Tomato (cultivar Moneymaker) leaf discs (1 cm diameter) were prepared and placed in 70% ethanol for 30 s with gentle swirling, then washed three times with 200 ml of sterile water. These leaf discs were placed on water agar with the adaxial surface facing upwards and allowed to air-dry in a sterile flow hood. Overnight bacterial cultures were adjusted to 10^8^ CFU ml^–1^ in 10 mM MgCl_2_. Drops (10 μl) of each strain were then inoculated on the adaxial surface of the leaf discs and allowed to air-dry. After 5 h, the leaf pieces were gently washed by placing each disc in 1 ml of sterile 0.85% NaCl solution and gently inverting 3–4 times to remove unattached cells. Three washed leaf discs were placed in 1 ml of sterile 0.85% NaCl, vortexed for 30 s to release adhered cells, followed by serial dilution and plating onto L agar with rifampicin (50 µg ml^–1^) and nystatin (20 µg ml^–1^) plates for CFU counting. Three such replicates for each strain and at least two independent experiments were performed. For *in vitro* leaf surface survival assays, tomato (cultivar Moneymaker) leaf discs (1 cm diameter) were prepared and inoculated with bacteria as above. At the respective time points, leaf discs were collected and placed in 1 ml of sterile 10 mM MgCl_2_, vigorously vortexed for 30 s to release cells, and CFU were counted following serial dilution.

### RNA isolation and qRT–PCR

Total RNA was extracted from cells grown on KB agar or SFM agar plates for 24 h at 28 °C. For low temperature treatment, 24-hour-old cultures were shifted from 28 °C to 8 °C and allowed to grow for another 24 h before collection. Cells scraped from plates were resuspended in 1 ml of RNA later and pelleted by centrifugation. RNA was isolated from pelleted cells using column capture (Qiagen RNeasy Mini Kit) following the manufacturer’s instructions. Purified RNA was subjected to additional DNase treatment (Turbo DNase, Ambion). RNA integrity was verified by agarose gel electrophoresis, and the absence of genomic DNA contamination was confirmed by a negative response to 16S rRNA gene amplification in PCRs using isolated RNA as template. cDNA was prepared from isolated RNA using Superscript II reverse transcriptase (Invitrogen) following the manufacturer’s instructions. Gene-specific primers were designed using the IDT primer quest tool, then quantitative reverse transcription–PCR (qRT–PCR) assays were conducted in a Bio-Rad CFX96 Touch RT-PCR machine using a SensiFAST™ SYBR® No-ROX Kit, with the following settings: 3 min at 95 °C and 50 cycles of 5 s at 95 °C, 10 s at 62 °C, and 10 s at 72 °C followed by a melting curve. Two independent RNA extractions and three technical replicates per extraction were assessed. *16S rRNA* and *gyrA* genes were used as internal controls for calculation of relative gene expression. Gene-specific primer sequences (nos 79–92) used in this study are listed in (Supplementary Table S1).

### TEM and imaging

*Pst* cells grown on KB agar for 24 h were harvested and fixed using a method described by (DeLucia *et al.* (2011). In brief, cells from agar were fixed using a mixture of 2.5% glutaraldehyde and 2% paraformaldehyde in 0.1 M sodium cacodylate buffer (CB), pH 7.4, for 2 h at room temperature. Chemically fixed samples were washed in 0.1 M CB and post-fixed with 1% osmium tetroxide (OsO4)–1.5% potassium ferricyanide {K_3_[Fe(CN)_ 6_]} for 1 h, washed in water three times, incubated in 1% aqueous uranyl acetate for 1 h, then dehydrated in ascending grades of ethanol (30, 50, 70, 95, and two changes of 100% ethanol, each for 1 h). Once dehydrated, the samples were gradually infiltrated with LR White resin (London Resin Company, Reading, UK) by successive changes of resin:ethanol mixes at room temperature (1:1 for 1 h, 2:1 for 1 h, 3:1 for 1 h, 100% resin for 1 h, then 100% resin for 16 h and a fresh change again for a further 8 h) then the samples were transferred into gelatine capsules full of fresh LR White and placed at 60 °C for 16 h to polymerize. The material was sectioned with a diamond knife using a Leica UC6 ultramicrotome (Leica, Milton Keynes, UK), and ultrathin sections of ~90 nm were picked up on 200 mesh copper grids which had been coated with pyroxylin and carbon. The sections were stained with 2% (w/v) uranyl acetate for 1 h and 1% (w/v) lead citrate for 1 min, washed in distilled water, and air-dried. The grids were viewed in a FEI Talos 200C transmission electron microscope (FEI UK Ltd, Cambridge, UK) at 200 kV and imaged using a Gatan OneView 4K×4K digital camera (Gatan, Cambridge, UK) to record DM4 files.

## Results

### Deletion of individual *Pst* polysaccharide loci has little effect on Arabidopsis infection

The *Pst* genome contains well-conserved predicted gene clusters for production of the polysaccharides alginate, Wss, Psl, α-glucan, and levan, as well as WapP/WaaP LPS biosynthetic clusters as per annotations in pseudomonas.com (Winsor *et al.*, 2016), summarized in Supplementary Figs S1–S6. Levan has been suggested to function as an apoplastic storage molecule (Laue *et al.*, 2006; Yu *et al.*, 2013) and is not implicated in biofilm or survival on leaf surfaces. Therefore, it was not studied further here. To probe the relationship between the remaining polysaccharide pathways and plant pathogenicity, we produced non-polar deletion mutants in key biosynthetic genes from each operon. Deletions in *algX-G* (Δ*alg*), *wssB-C* (Δ*wss*), and *pslD*-*E* (Δ*psl*), alongside a triple mutant of all three clusters (Δ*EPS*) have been described previously (Lammertz *et al.*, 2019). *Pst* has a similar arrangement of trehalose and α-glucan biosynthetic genes to *P. aeruginosa* PA01 (Woodcock *et al.*, 2021), and we predict that the pathway is likely to function similarly in both species (Supplementary Figs S5, S6, S7. Consequently, we produced mutants of the *treS* (*ΔPSPTO2760-62*) and *treY/Z* (Δ*PSPTO3125-3130*) operons. Finally, we deleted the conserved, putative LPS kinase gene *wapQ* (*PSPTO4998*) (Supplementary Figs S1, S8). *wapQ* can be deleted in *P. aeruginosa*, where its disruption induces phenotypes consistent with LPS disruption (Walsh *et al.*, 2000), but has relatively minor effects on bacterial fitness, unlike many other LPS biosynthetic genes. Despite this, *wapQ* is highly conserved between the LPS biosynthetic operons of *Pseudomonas* spp. (Kutschera *et al.*, 2021).

**Fig. 1.**
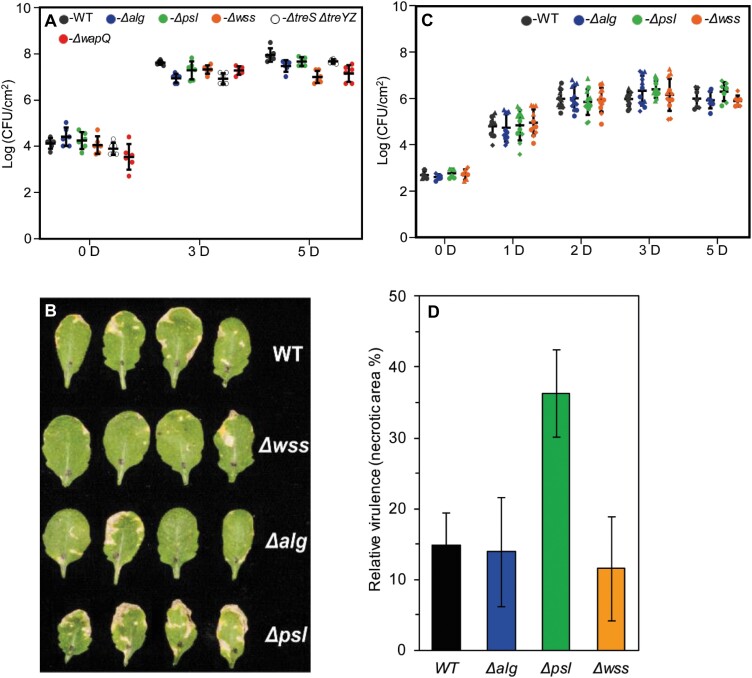
Deletion of individual polysaccharide loci has little effect on *Pst* Arabidopsis infections (A) Bacterial load following spray infection at different days post-infection [D]. Means ±SD from biological duplicates and technical triplicates are shown. (B) *Arabidopsis thaliana* Col-0 leaves 5 d post-infiltration with different *Pst* mutants. Similar results were obtained in three biologically independent experiments and a representative picture is shown. (C) Bacterial load post-infection following infiltration at different days post-infection [D]. Means ±SD from three biologically independent experiments are presented in each case. Values from independent experiments are represented using different shapes. (D) Relative virulence calculated based on necrotic area (from leaves in B) 5 d post-infiltration.

To investigate the role of individual polysaccharides in plant infection, we conducted a series of infection experiments in *A. thaliana* Col-0 (Col-0) plants. No significant differences were observed between any of the *Pst* single mutant strains for spray infections (Fig. 1A). Previous research has highlighted the importance of the EPS alginate in apoplastic, but not epiphytic, survival in *P. syringae* B728a (Helmann *et al.*, 2019). This suggests that bypassing the initial stages of infection may uncover differences between our mutants. To test this, we conducted leaf infiltration assays with our three *Pst* EPS single mutants. However, while a slight increase in necrosis symptoms was observed for leaves infected with the Δ*psl* mutant (Fig. 1B), no significant difference in bacterial load versus the wild type was seen for any of the single EPS mutants tested (Fig. 1C).

### Wss, alginate, Psl, and α-glucan production are regulated by cyclic-di-GMP and nutrient availability

**Fig. 2.**
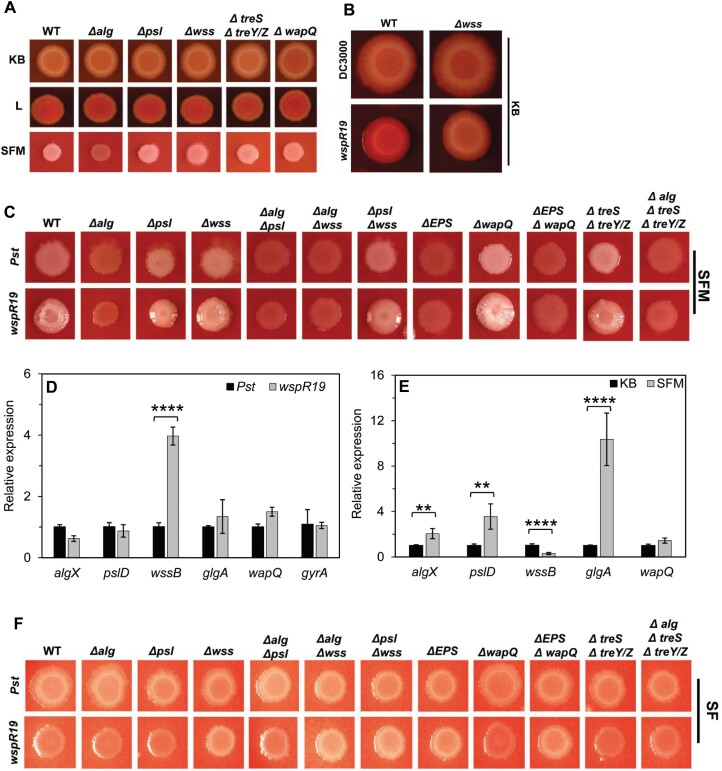
Wss, alginate, Psl, and α-glucan production are stimulated by cyclic-di-GMP and nutrient availability. (A) Phenotypes of mutant strains on CR-supplemented King’s B (KB), L medium (L), and soy flour mannitol medium (SFM) agar plates. (B) Effect of high cyclic-di-GMP on *Pst* colony morphology at 28 °C on CR-supplemented KB. (C) Colony morphology of *Pst* strains on CR-supplemented SFM. Strains with natural levels of cyclic-di-GMP (*Pst*); strains with high c-di-GMP (*wspR19*). (D) Quantitative real-time PCR analysis of the expression of polysaccharide-producing genes in the wild type (WT; *Pst*, black) and in the presence of high levels of cyclic-di-GMP (*wspR19*, grey). (E) Quantitative real-time PCR analysis of the expression of polysaccharide-producing genes for WT *Pst* grown on KB agar (black) and SFM agar (grey). Error bars represent means ±SD with *n*=2 (three technical replicates each) for (D) and (E). Significance as determined by Student’s *t*-test (∗∗*P*<0.005 and ∗∗∗∗*P*<0.0001). (F) Colony morphology of *Pst* strains on CR-supplemented soy flour (SF) medium. Similar results were obtained in two independent experiments, and a representative picture is shown.

To understand why the *Pst* polysaccharide loci are apparently dispensable for plant infection, we tested their expression and production in response to a variety of intracellular and external signals. In general, disruption of individual polysaccharide loci did not lead to marked differences in *Pst* morphology for colonies grown on different nutrient media, with a few exceptions (Fig. 2A). On SFM, the distinctive, alginate-dependent white mucoid colony phenotype was absent in the Δ*alg* mutant. Deletion of Δ*treS* Δ*treY/Z* produced noticeably thicker, more mucoid white colonies on SFM, suggesting a possible increased production of alginate in the absence of α-glucan. Deletion of *wapQ* produced a distinctive phenotype on KB CR agar, with a dark halo surrounding the central zone of the *Pst* colony and higher CR binding at the centre of the colony on L agar with CR (Fig. 2A; Supplementary Fig. S9A). CR is an amyloid dye that is widely used in studying production of EPS molecules due to its strong binding to certain polysaccharides. The transient CR binding in the centre of the colony by Δ*wapQ* suggests that WapQ plays a role in altering the *Pst* cell surface, possibly via modification of LPS. However, the absence of WapQ did not result in the visible accumulation of polysaccharides inside the cells, as was observed for the WaaP kinase of *P. aeruginosa* (DeLucia *et al.*, 2011) (Supplementary Fig. S9B, C).

The second messenger cyclic-di-GMP stimulates EPS production in many bacterial species including *Pst* (Jenal *et al.*, 2017; Perez-Mendoza *et al.*, 2019). To examine how cyclic-di-GMP levels affect *Pst* polysaccharide production, we transformed our mutants with the plasmid pBBR-*wspR19*, which elevates cyclic-di-GMP levels by ~15-fold in *Pst* (Pfeilmeier *et al.*, 2016b). Curiously, unlike with many *Pseudomonas* strains (Malone *et al.*, 2010), we saw little impact of cyclic-di-GMP overproduction on colonies grown on KB agar at 28 °C, besides a slight increase in CR binding that was absent in the Δ*wss* background (Fig. 2B; Supplementary Fig. S10A). Conversely, growth on SFM led to the formation of mucoid, wrinkly colonies upon *wspR19* expression (Fig. 2C). Two polysaccharide gene deletions markedly affected this colony morphology. First, *wspR19+*Δ*psl* mutants produced smooth, mucoid colonies, supporting a role for Psl in maintaining the architecture of wrinkly colonies on SFM. Second, *alg* disruption abolished both mucoidy and the wrinkly phenotype in all backgrounds, implicating alginate as the primary structural EPS for *Pst* on SFM (Fig. 2C).

These results were supported by qRT–PCR of the *EPS* genes. *wssB* mRNA abundance increased markedly upon *wspR19* expression in *Pst* grown on KB agar, while the other tested genes were unaffected (Fig. 2D). However, a comparison of wild-type *Pst* grown on KB and SFM agar showed significantly higher *algX*, *pslD*, and *glgA* mRNA abundance, and reduced levels of *wssB* mRNA for colonies on SFM, suggesting that expression of these EPS loci is strongly dependent on nutrient cues from the environment (Fig. 2E). The cyclic-di-GMP-dependent, alginate-linked mucoid phenotype disappeared in the absence of mannitol; that is; for strains grown on SF agar, suggesting that mannitol is a key nutrient for alginate production. Curiously, *Pst wspR19+* strains lacking the complete Wss produced white slightly mucoid colonies on SF plates. This phenotype was independent of alginate, Psl, or WapQ, suggesting that another, unknown polysaccharide may be up-regulated under these conditions (Fig. 2F).

### Wss, Psl, and α-glucan production are stimulated, while alginate is suppressed, by low temperature

**Fig. 3.**
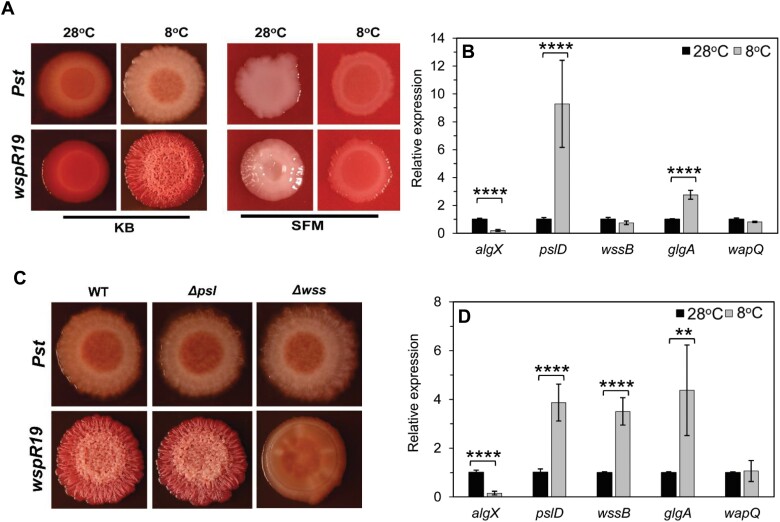
Wss, Psl, and α-glucan production are stimulated by low temperature. (A) Effect of temperature on *Pst* colony morphology on CR-supplemented media. Wild-type (WT) *Pst* and *Pst wspR19* grown on KB and SFM at 28 °C and 8 °C. (B) qRT–PCR analysis of polysaccharide genes in WT *Pst* grown on KB agar at 28 °C (black) and 8 °C (grey). (C) Colony morphology of the WT, Δ*psl*, and Δ*wss* on KB agar after 5 d of cold treatment at 8 °C. Strains with high cyclic-di-GMP (*wspR19*) are shown. (D) qRT–PCR analysis of polysaccharide genes in *Pst wspR19* grown on KB agar at 28 °C (black) and 8 °C (grey). Values represented are means ±SD with *n*=2 (three technical replicates each). Significance was determined by Student’s *t*-test (∗∗*P*<0.005 and ∗∗∗∗*P*<0.0001). (A and C) Similar results were obtained in two independent experiments and a representative image is shown.

Next, we examined the impact of temperature on polysaccharide gene expression and the associated changes in colony morphology. Switching the temperature from 28 °C to 8 °C produced relatively subtle changes in wild-type *Pst* but led to the formation of dry, wrinkled colonies upon *wspR19* expression on KB agar (Fig. 3A). The switch from 28 °C to 8 °C led to significant increases in mRNA abundance for *pslD* and *glgA* and reduced levels of *algX* (Fig. 3B). A similar pattern was seen for *Pst wspR19+*, with the exception of *wssB*, where a greater increase in mRNA abundance was seen at 8 °C than at 28 °C (compare Figs 2D and 3B–D).

The wrinkled colony phenotype on KB was dependent on Wss alone (Fig. 3C). Conversely, growth at 8 °C saw an almost complete abolition of the alginate-dependent, wrinkled mucoid phenotype on SFM plates (Fig. 3A; Supplementary Fig. S10A, B). This was further supported by the decreased levels of *algX* mRNA seen at low temperature (Fig. 3B–D). The Wss-dependent wrinkled morphology did not manifest on SFM plates at 8 °C, suggesting that Wss production is nutrient dependent in *Pst* in a similar manner to Psl and alginate (Fig. 3A; Supplementary Fig. S10B).

### α-Glucan and alginate production are stimulated by global carbon storage regulators and surface association

**Fig. 4.**
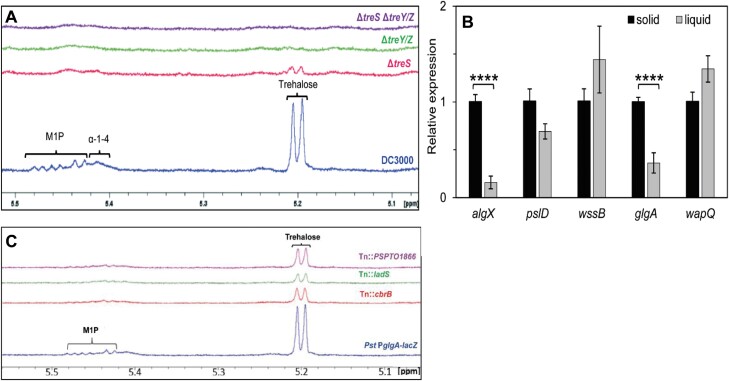
α-Glucan and alginate biosynthesis are stimulated by global carbon storage regulators and surface association. (A) ^1^H-NMR spectra for *Pst* wild type (WT; blue), Δ*treS* (pink), Δ*treY/Z* (green), and a double mutant Δ*treS* Δ*treY/Z* (purple). Peaks corresponding to key metabolites are labelled: maltose 1-phosphate (M1P), α-glucan (α-1,4), α-1,1-probable terminal linkage of maltooligosyltrehalose (trehalose). (B) qRT–PCR analysis of polysaccharide genes in WT *Pst* grown on KB agar (solid) versus KB liquid (liquid). Values represented are means ±SD with *n*=2 (three technical replicates each). Significance was determined by Student’s *t*-test (∗∗∗∗*P*<0.0001. (C) ^1^H-NMR spectra for the *Pst* P*glgA-lacZ* reporter strain and selected *glgA* regulatory Tn mutants. Peaks corresponding to key metabolites are labelled as in (A).

The *treS* and *treY/Z* operons have previously been implicated in *Pst* leaf surface survival (Freeman *et al.*, 2010), suggesting that their expression is likely to be most relevant during epiphytic growth. To investigate this further, we first determined the abundance of key metabolites in *Pst* by ^1^H-NMR spectroscopy. Wild-type *Pst* grown in M9 medium accumulated trehalose and maltose 1-phosphate (M1P) to 0.13 ± 0.03% and 0.30 ± 0.03% of cellular dry weight, respectively (Table 2), alongside a broad NMR peak at 5.41 ppm corresponding to α-glucan (Fig. 4A). Deletion of the *treS* operon alone (*glgE*, *treS/pep2*, and *glgB*) not only blocked the production of M1P and α-glucan, as expected, but also led to less trehalose being detected (0.04 ± 0.01%) compared with the wild type. Deletion of *glgA*, *treZ*, *malQ*, *treY*, and *glgX* (Δ*treY/Z*) resulted in no detectable metabolites from these pathways as expected (Fig. 4A; Table 2), whether or not the other operon was deleted.

**Table 2.** Concentrations of trehalose and M1P produced by *Pst* strains

Strain	Trehalose (%)	M1P (%)	Presence of α-glucan
WT	1.33 ± 0.14	0.43 ± 0.15	+
Δ*treS*	0.04 ± 0.01	ND	–
Δ*treY/Z*	ND	ND	–
Δ*treS* Δ*treY/Z*	ND	ND	–

Metabolites are presented as percentages of cellular DW ±SE (*n* ≥ 2). ND indicates no measurable metabolite. Presence or absence of α-glucan is indicated by + or –, respectively.

Next, we used qRT–PCR to measure mRNA abundance for the different polysaccharide genes for *Pst* cells grown on a solid agar surface and in liquid media. Normalized to the *gyrA* internal control, the mRNA level for the α-glucan synthase gene *glgA* was significantly higher in surface-grown compared with liquid-grown *Pst* cells (Fig. 4B). Interestingly, the same pattern of increased mRNA abundance in surface-grown cells was also seen for *algX*, while no differences were seen for the other tested genes.

To further investigate the regulation of *Pst* trehalose/α-glucan gene expression, the 500 bp region upstream of the *glgA* start codon was cloned upstream of *lacZ* and incorporated into the *Pst* chromosome at the neutral *att*::Tn*7* site. The resulting strain (*Pst* P*glgA*-*lacZ*) produced blue colonies on XGal+IPTG plates and was then used to conduct a transposon mutagenesis screen for regulators of *glgA* transcription. Once false positives/negatives and likely indirect hits had been discarded, we identified promising transposon insertions in three regulatory loci: the response regulator *cbrB*, the histidine kinase gene *ladS*, and a predicted TetR family transcriptional regulator, *PSPTO1866*. Transposon insertion led to light blue colonies in each case, suggesting that these regulators may function as activators of *glgA* expression.

To test the effects of gene disruption on trehalose and α-glucan biosynthesis, the *Pst* water-soluble metabolome was analysed for the three transposon mutants. No significant difference was observed between the levels of analysed metabolites in the soluble metabolomes of wild-type *Pst* and *Pst* P*glgA*-*lacZ*. Tn::*PSPTO1866* showed a small, but not statistically significant (*P*=0.06) reduction in trehalose and no change in M1P abundance compared with *Pst* P*glgA*-*lacZ.* Conversely, the metabolomes of Tn::*cbrB* and Tn::*ladS* showed significant decreases in both trehalose and M1P levels (Fig. 4C; Table 3), supporting roles for the global carbon utilization and chronic/acute lifestyle regulatory proteins LadS (and by extension the Gac/Rsm pathway) and CbrB in stimulating trehalose and α-glucan gene expression (Sonnleitner *et al.*, 2009; Grenga *et al.*, 2017).

**Table 3.** Concentrations of trehalose and M1P produced by the *Pst* P*glgA-lacZ* strain and mutants generated by transposon random mutagenesis

Strain	Trehalose (%)	M1P (%)
*Pst*-P*glgA-lacZ*	1.68 ± 0.19	0.20 ± 0.03
Tn::*cbrB*^*a*^	0.41	0.11
Tn::*ladS*	0.31 ± 0.07∗	0.05 ± 0.02
Tn::*PSPTO1866*	0.82 ± 0.21^†^	0.26 ± 0.04

Metabolites are presented as percentages of cellular DW ±SE (*n*≥2).

^*a*^Indicates *n*=1.

Statistical significance compared with parent strain, ∗*P*<0.05 and ^†^*P*>0.05 determined by Student’s *t*-test.

### Disruption of trehalose, α-glucan, and alginate production leads to stress sensitivity and compromised plant infection

**Fig. 5.**
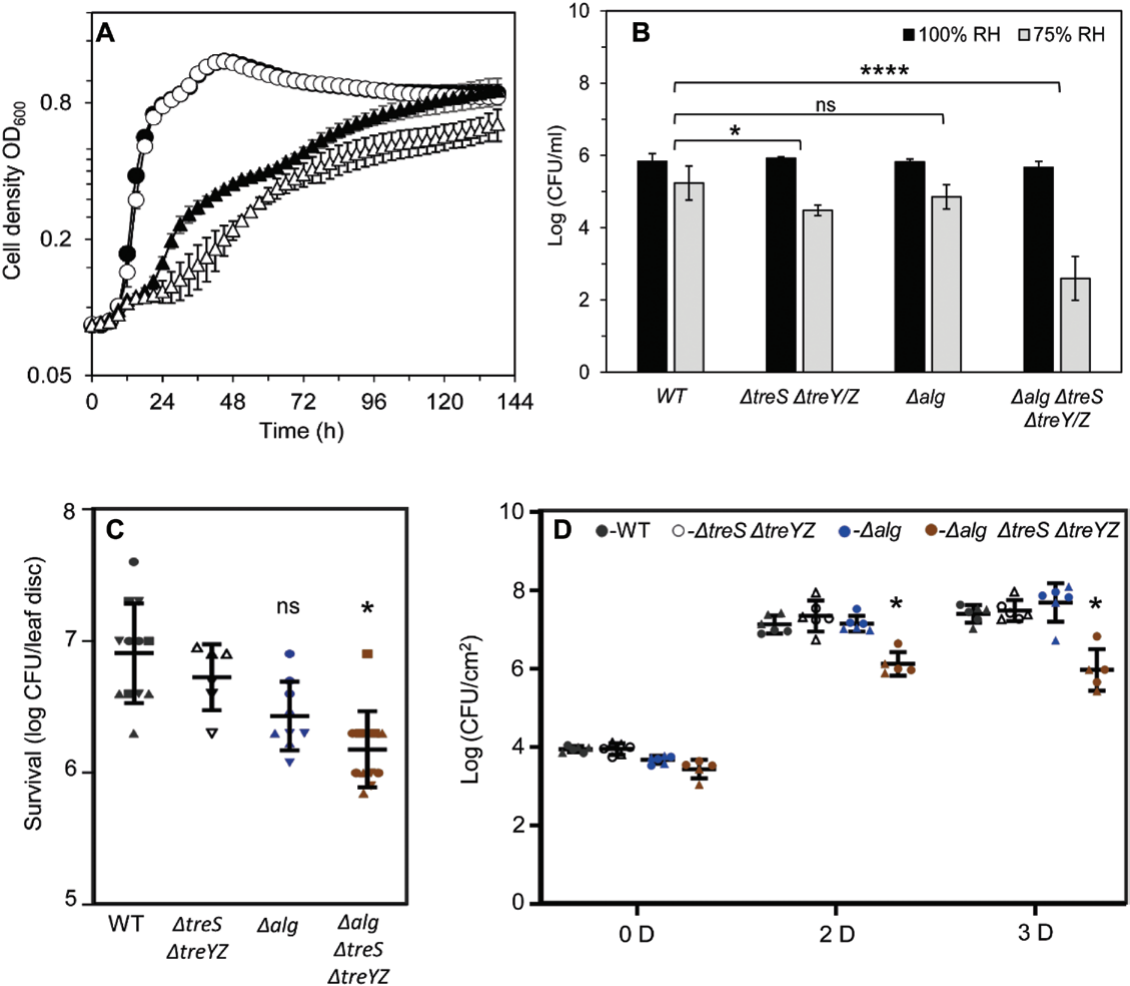
Disruption of trehalose, α-glucan, and alginate production leads to stress sensitivity and compromised plant infection. (A) Growth of the wild type (WT) and Δ*treS* Δ*treY/Z* in M9 medium (circles), WT (filled), Δ*treS* Δ*treY/Z* (open), and in M9 medium supplemented with 0.35 M NaCl (triangles), WT (filled), Δ*treS* Δ*treY/Z* (open). Similar results were obtained for three biologically independent experiments. Data shown are the means ±SD of three biological replicates with two technical replicates each. (B) Desiccation stress survival assays for strains exposed to 100% relative humidity (black) and 75% (grey) for 2 h. Data shown are means ±SD of three biological replicates. (C) Surface survival assay on tomato leaf discs after 24 h. Recovered CFU after 24 h for selected mutant strains are presented. Similar results were obtained in at least two independent experiments. Means ±SD are presented, with values from independent experiments and triplicates of each measurement represented with a different shape in the figure. (D) Bacterial load following spray infection at different days post-infection [D]. WT *Pst* (black), Δ*treS* Δ*treY/Z* (white), Δ*alg* (blue), and Δ*treS* Δ*treY/Z* Δ*alg* (brown). Means ±SD are presented. with values from two independent experiments shown using a different shape. In each case, significance was determined by Student’s *t*-test (∗*P*<0.05, ∗∗∗∗*P*<0.0001, and ns not significant).

To investigate the roles of trehalose and α-glucan in *Pst* during osmotic stress, wild-type *Pst* and Δ*treS* Δ*treY/Z* were grown in the presence and absence of 0.35 M NaCl in M9, a defined medium. While both strains exhibited a longer lag phase and attenuated growth rate when cultured in 0.35 M NaCl, the Δ*treS* Δ*treY/Z* mutant showed substantially increased osmotic sensitivity relative to the wild type as expected, with the production of trehalose being blocked (Fig. 5A). Next, we analysed the relative desiccation stress tolerance of Δ*treS* Δ*treY/Z* and wild-type *Pst* as described previously (Woodcock *et al.*, 2021). The CFU recovered after exposure to 100% and 75% relative humidity were counted, analysed using linear mixed modelling, and represented as predicted means of log_10_(CFU ml^–1^). The sensitivity of a strain to reduced humidity was calculated as the difference between its mean log_10_(CFU ml^–1^) at 100% and at 75% relative humidity. A greater response to lower relative humidity of a mutant compared with the wild type translates to a more desiccation-sensitive strain. Following incubation of bacterial spots on filter discs at 100% relative humidity, 5.83 log_10_(CFU ml^–1^) wild-type cells were recovered against 5.23 log_10_(CFU ml^–1^) at 75% relative humidity, equating to a desiccation response of ~0.60 log_10_(CFU ml^–1^) (Fig. 5B). The equivalent values for Δ*treS* Δ*treY/Z* were 5.92 (100% relative humidity) and 4.47 (75% relative humidity) log_10_(CFU ml^–1^), giving a desiccation response of ~1.50 log_10_(CFU ml^–1^) (Fig. 5B). Based on our recent analysis of PA01 (Woodcock *et al.*, 2021), it is likely that this desiccation-sensitive phenotype is linked to the loss of α-glucan production.

Alginate has also previously been associated with bacterial stress tolerance and epiphytic fitness (Yu *et al.*, 1999; Helmann *et al.*, 2019). Based on the apparent link between their colony morphology phenotypes on SFM plates and co-expression on a solid surface (KB-agar), we investigated the role of alginate in protecting *Pst* during desiccation. As the Δ*alg* strain yielded a desiccation response of 0.96 log_10_(CFU ml^–1^), which was not significantly different to the response of wild-type *Pst* when analysed using linear mixed modelling (Fig. 5B), we tested whether α-glucan and alginate may exhibit functional redundancy in *Pst.* An alginate+α-glucan mutant (Δ*alg* Δ*treS* Δ*treY/Z*) was generated and exposed to desiccation stress. This strain yielded a highly significant (*P*≤0.0001) desiccation response of 3.1 log_10_(CFU ml^–1^) (Fig. 5B). Furthermore, the Δ*alg* Δ*treS* Δ*treY/Z* strain showed reduced epiphytic survival on tomato leaf discs, while no significant difference was observed for other tested strains after 24 h (Fig. 5C). This suggests that alginate and α-glucan functionally complement each other during desiccation stress response on plant leaves.

Next, we investigated whether the interaction of trehalose/α-glucan and alginate is also relevant during plant infection. Col-0 plants were spray infected with the different Δ*alg* and Δ*treS* Δ*treY/Z* mutants monitored over 3 d growth without watering. In agreement with our previous assay (Fig. 1A), a lack of neither alginate nor trehalose/α-glucan alone affected the course of infection. However, we saw a significant decrease in bacterial cell counts for infections with Δ*alg* Δ*treS* Δ*treY/Z* both 2 d and 3 d post-infection (Fig. 5D; Supplementary Fig. S11A). Together, our results suggest that alginate and α-glucan work together in *Pst* to mediate both desiccation stress tolerance and plant infectivity. Interestingly, no significant difference in bacterial colonization was observed either when plants are infiltrated or if plants were watered during the course of infection (Supplementary Figs S12A, S13). This supports the hypothesis that alginate and α-glucan play roles in surviving the water stress during epiphytic survival.

### Disrupting *wapQ* and EPS alters *Pst* leaf adhesion and compromises plant infection

**Fig. 6.**
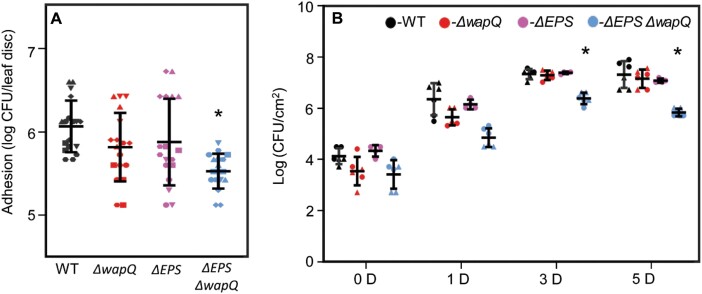
Disrupting EPS and the putative LPS kinase WapQ alters *Pst* leaf adhesion and compromises plant infection. (A) Adhesion to tomato leaf surfaces. The graph denotes the number of cells adhered to leaf surfaces after 5 h. Means ±SD are presented, with values from two independent experiments and triplicates of each measurement represented with a different shape in the figure. (B) Bacterial load following spray infection at different days post-infection [D]. Wild-type (WT) *Pst* (black), Δ*wapQ* (red), Δ*EPS* (purple), and Δ*EPS* Δ*wapQ* (blue). Similar results were obtained in two biologically independent experiments and means ±SD are presented, with values from two independent experiments and triplicates of each measurement represented with a different shape in the figure. In each case, ∗*P*<0.05 determined by Student’s *t*-test.

Several studies have suggested links between the LPS and EPS pathways in *Pseudomonas* spp. (McDonald *et al.*, 2009). Given the lack of impact of single gene deletions on Col-0 infection and the complementarity observed between alginate and α-glucan, we tested the effect of disrupting both *wapQ* and the EPS biosynthesis loci on leaf surface interaction and plant infection. Following initial trial assays, Arabidopsis leaves proved too small for reliable measurements of surface association, so tomato leaves (cultivar Moneymaker) were used to examine *Pst* adhesion to and survival on leaf surfaces. In leaf disc adhesion assays (Arrebola *et al.*, 2015), the Δ*EPS* Δ*wapQ* mutant showed a statistically significant reduction (*P*<0.05) in adhesion to tomato leaf discs compared with the other tested strains (Fig. 6A). Consistent with this, the Δ*EPS* Δ*wapQ* mutant displayed visibly compromised disease phenotypes on Col-0 plants, alongside significantly lower bacterial counts relative to wild-type *Pst*, the ΔEPS triple mutant, or Δ*wapQ* after 3 d and 5 d upon spray infection (Fig. 6B; Supplementary Fig. S11B). However, no significant differences in bacterial counts were observed upon infiltration (Supplementary Fig. S12B). This suggests that the altered surface attachment phenotype seen with this mutant translates into compromised plant infections.

## Discussion

Phyllosphere-colonizing bacterial pathogens such as *P. syringae* face an array of environmental challenges on external plant surfaces (Wu *et al.*, 2012; Djonovic *et al.*, 2013; Freeman *et al.*, 2013; Pfeilmeier *et al.*, 2016b). Phytopathogenic bacteria must be able to tolerate rapid shifts in humidity and osmotic pressure, to adhere to leaf surfaces under mechanical disturbance, and to respond effectively to changes in temperature and nutrient availability, among other cues. Systems that facilitate bacterial survival and persistence in the face of these challenges thus play important roles in enabling infection (Pfeilmeier *et al.*, 2016a). Based on gene/protein homologies, EPS pathways and cell envelope LPSs are ubiquitous in *P. syringae* genomes (Winsor *et al.*, 2016) and are among the first bacterial molecules to interact with the host plant (Mhedbi-Hajri *et al.*, 2011; King and Roberts, 2016). Polysaccharide biosynthesis has been linked to epiphytic survival, stress tolerance, and biofilm formation in various phytopathogenic bacteria (Pfeilmeier *et al.*, 2016a). It has also been shown that alginate might play role in apoplastic survival in *P. syringae* B728a (Helmann *et al.*, 2019). Furthermore, as major components of the biofilm matrix, polysaccharides play important roles not just in leaf surface survival but also during the transition to more acute stages of pathogenicity (Yu *et al.*, 1999; Dunger *et al.*, 2007; Pfeilmeier *et al.*, 2016b; Xin *et al.*, 2018).

**Fig. 7.**
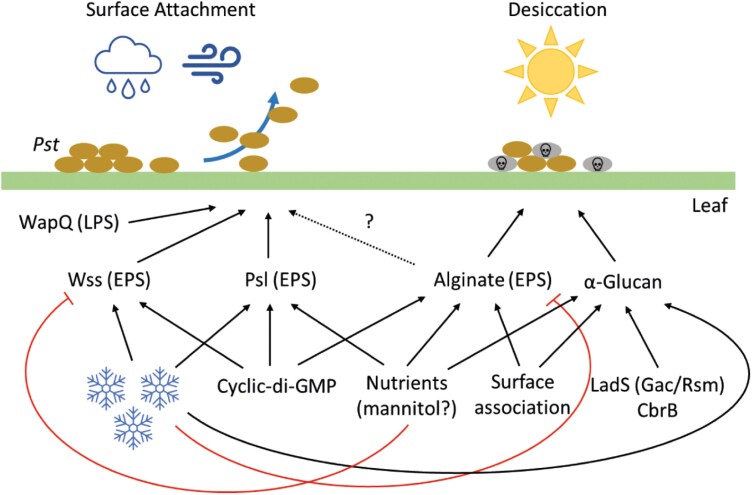
A model for polysaccharide deployment during plant infection by *P. syringae Pst*. Positive or stimulatory interactions are denoted by black arrows. Negative/suppressive interactions are shown by red arrows. An uncertain interaction is indicated with a dashed arrow. Blue snowflakes denote low temperature, brown ovals indicate *Pst* cells, and grey ovals indicate dying cells.

In this study, we examine the regulation of and interactions between five major polysaccharide pathways in *Pst* and determine their importance to plant infection and epiphytic survival. While EPS and LPS pathways have been implicated in phytopathogen virulence, stress response, and biofilm formation (Pfeilmeier *et al.*, 2016a), previous studies have generally focused on the impact of individual polysaccharides (Dunger *et al.*, 2007; Arrebola *et al.*, 2015; Heredia-Ponce *et al.*, 2020). The importance of interactions between the different polysaccharide pathways for plant infection and epiphytic survival is currently poorly understood. Our results suggest that polysaccharide production is tightly regulated and coordinated in *Pst*, with deployment of each polysaccharide system dependent on a number of shared environmental cues (Fig. 7). We observed a substantial degree of regulatory and functional redundancy: each external input stimulated multiple polysaccharide pathways, and infection/survival phenotypes were only observed for *Pst* mutants missing several coordinating pathways.

Cyclic-di-GMP conditionally stimulates production of all three *Pst* EPS molecules. However, unlike in other *Pseudomonas* spp. where increased cyclic-di-GMP levels lead to constitutive EPS production and wrinkled, aggregative colony morphologies (Malone et al., 2007, 2010), cyclic-di-GMP overproduction in *Pst* does not lead to major changes in colony morphology under standard laboratory conditions. Rather, *Pst* EPS production is highly dependent on external environmental cues, with production of the two major structural EPS molecules—Wss and alginate—stimulated by low temperature and changes to nutrient availability respectively. In each case, the formation of Wss- or alginate-driven wrinkled colonies requires both increased cyclic-di-GMP production and the correct growth environment. The two phenotypes are mutually exclusive, with Wss linked to growth on KB medium at low temperatures and alginate to SFM and surface association, but with little/no effect seen for gene deletions in the other condition.

Psl, which maintains the architecture of mucoid, wrinkly colonies on SFM plates, is stimulated both by low temperature and a switch to SFM, suggesting that Psl is linked to both the alginate- and Wss-mediated lifestyles. A central role for Psl in mediating the *Pst* transition from an epiphytic, biofilm lifestyle to more acute pathogenesis was supported by the apparent increase in disease symptoms seen for the Δ*psl* mutant during Col-0 leaf infections. Similar observations have been reported for a *Salmonella enterica* cellulose mutant, and upon Psl or cellulose disruption in *P. syringae* UMAF0158 (Pontes *et al.*, 2015; Heredia-Ponce *et al.*, 2020). The mechanism by which Psl suppresses disease symptoms during infection, and the relevance of this for bacterial fitness in the plant environment remain to be determined.

Expression of *glgA*, the first gene of the *treY/Z* operon in *Pst*, is stimulated by surface association, SFM, and the global regulatory proteins CbrB and LadS. The response regulator CbrB and its cognate signal kinase CbrA control carbon metabolism, biofilm formation, and stress tolerance (Nishijyo *et al.*, 2001; Amador *et al.*, 2010) by inducing expression of RpoN-dependent genes such as the sRNA *crcZ* (García-Mauriño *et al.*, 2013), which acts to antagonize the carbon catabolite repressor Crc (Moreno *et al.*, 2009). The Gac/Rsm system controls processes including biofilm, motility, virulence, and the stress response at the level of mRNA translation (Brencic *et al.*, 2009; Chambers and Sauer, 2013). In *Pseudomonas* spp., the Gac/Rsm system is controlled by a series of accessory sensor kinases including LadS, which acts as a positive regulator of GacAS activity (Chambonnier *et al.*, 2016).

Trehalose/α-glucan and alginate production appears to be subject to a shared regulatory hierarchy in *Pseudomonas* spp. CbrA/CbrB has been implicated in controlling alginate production in *P*. *fluorescens* (Ertesvåg *et al.*, 2017), and ChIP-seq analysis of the *P. putida* genome identified a CbrB-binding site in the *algD* promoter (Barroso *et al.*, 2018). Similarly, the Gac/Rsm system regulates alginate production in both *Azotobacter* and *Pseudomonas* (Manzo *et al.*, 2011; Romero *et al.*, 2018), with the anti-sigma factor gene *mucA* an mRNA target for Rsm proteins in *P*. *aeruginosa*. CbrA/CbrB and LadS are positive regulators of both alginate and trehalose/α-glucan biosynthesis (Ertesvåg *et al.*, 2017; Romero *et al.*, 2018), which makes sense in the context of their coordinated roles in protecting the cell from desiccation stress. Further research is needed to determine the extent to which the well-established alginate regulon (Wozniak and Ohman, 1994) also controls trehalose and α-glucan biosynthesis.

Trehalose/α-glucan, and potentially also alginate, individually contribute to *Pst* desiccation stress tolerance (Fig. 5B), although the effects on survival or infectivity of disrupting either pathway alone were minimal. However, we saw evidence for a substantial degree of functional redundancy between the two pathways, with trehalose/α-glucan/alginate triple mutants becoming highly sensitive to desiccation stress under laboratory conditions. This *in vitro* desiccation sensitivity translated to decreased epiphytic surface survival (Fig. 5C) and compromised plant infection when plants are grown in dry conditions (i.e. without watering; Fig. 5D). Although *Pst* is believed to be a weak epiphyte, our surface survival assays showed that a significant number of viable *Pst* cells remain on the surface of leaf discs even after 24 h of incubation. Our results therefore show that epiphytic survival is an important virulence determinant even for relatively weak epiphytes such as *Pst*. We also observed functional redundancy between the WapQ putative LPS kinase and the *Pst* EPS pathways. Leaf attachment and plant infection were unaffected for Δ*wapQ*, or upon disruption of the *wss*, *psl*, and *alg* operons. However, disruption of all four operons together led to significantly reduced leaf attachment and compromised Col-0 infection (Fig. 6A, B).

The emerging picture from our research is that *Pst* uses the coordinated deployment of distinct sets of polysaccharides to address different environmental challenges (Fig. 7). The first of these, desiccation and osmostress response, are addressed by the deployment of alginate, trehalose, and α-glucan in response to surface contact and changes to nutrient availability, under the control of the Gac/Rsm and CbrA/B regulatory pathways. Conversely, when attachment to leaf surfaces is a high priority, EPS pathways and particularly Wss are up-regulated in response to reduced temperatures and under the control of cyclic-di-GMP. These two responses appear to be mutually exclusive, with conditions that stimulate Wss production repressing the production of alginate, and vice versa.

Our data also suggest that in the context of these broad regulatory groups, *Pst* fine-tunes the deployment of individual polysaccharides to create an optimal response to the environment. For example, *glg* and *alg* regulation are closely aligned, except at low temperatures, where *alg* mRNA is reduced but *glgA* levels increase. Similarly, Psl appears to contribute to both alginate and Wss biofilm formation and is stimulated by SFM and low temperatures. Finally, cyclic-di-GMP signalling appears to stimulate several *Pst* polysaccharide pathways, although transcription-level regulation is only apparent for the *wss* locus. Further research is needed to fully understand the regulatory underpinnings of *Pst* polysaccharide deployment during plant infection.

## Supplementary data

The following supplementary data are available at *JXB* online.

Fig. S1. Mutagenesis of EPS-producing genes and a putative *wapQ* gene.

Fig. S2. Comparison of proteins encoded by alginate gene clusters.

Fig. S3. Comparison of proteins encoded by cellulose/*wss* gene clusters.

Fig. S4. Comparison of proteins encoded by Psl gene clusters.

Fig. S5. Comparison of proteins encoded by trehalose/α-glucan gene cluster-1.

Fig. S6. Comparison of proteins encoded by trehalose/α-glucan gene cluster-2.

Fig. S7. A scheme representing the pathway for the production of α-glucan in *Pst*.

Fig. S8. Comparison of WapQ protein sequences in pathovars of *P. syringae*.

Fig. S9. Effect of the absence of WapQ on *Pst* morphology.

Fig. S10. Effect of low temperature on colony phenotype of different mutant strains.

Fig. S11. Disease symptoms on Col-0 plants post-spray infection.

Fig. S12. Absence of alginate, α-glucan, EPS, and WapQ has no effect in infection upon infiltration.

Fig. S13. Absence of alginate and α-glucan has no effect on plant infection by *Pst* when plants are watered.

Table S1. List of primers used in this study.

erab550_suppl_supplementary_figures_S1-S13

## Data Availability

All data supporting the findings of this study are available within the paper and within its supplementary data published online. The data supporting the findings of this study and raw data used for making the graphs are available from the corresponding author, Jacob Malone, upon request.

## Abbreviations

CFU: colony-forming units
CR: Congo red dye
cyclic-di-GMP: bis-(3ʹ-5ʹ)-cyclic dimeric GMP
EPS: exopolysaccharide
KB medium: King’s B medium
LPS: lipopolysaccharide
M1P: maltose 1-phosphate
OD_600_: optical density at λ 600 nm
*Pst*: *Pseudomonas syringae* pv. *tomato* DC3000
SFM: soy flour mannitol medium
