# Supplementary material for: Pseudomonas syringae addresses distinct environmental challenges during plant infection through the coordinated deployment of polysaccharides
Source: J Exp Bot. 2021 Dec 14;73(7):2206–21. doi: 10.1093/jxb/erab550 (PMC8982409; doi:10.1093/jxb/erab550)
Supplement: erab550_suppl_supplementary_figures_S1-S13 [file erab550_suppl_supplementary_figures_s1-s13.pdf]

Figure S1. Genome context and mutagenesis of EPS producing genes and a putative *wapQ gene*

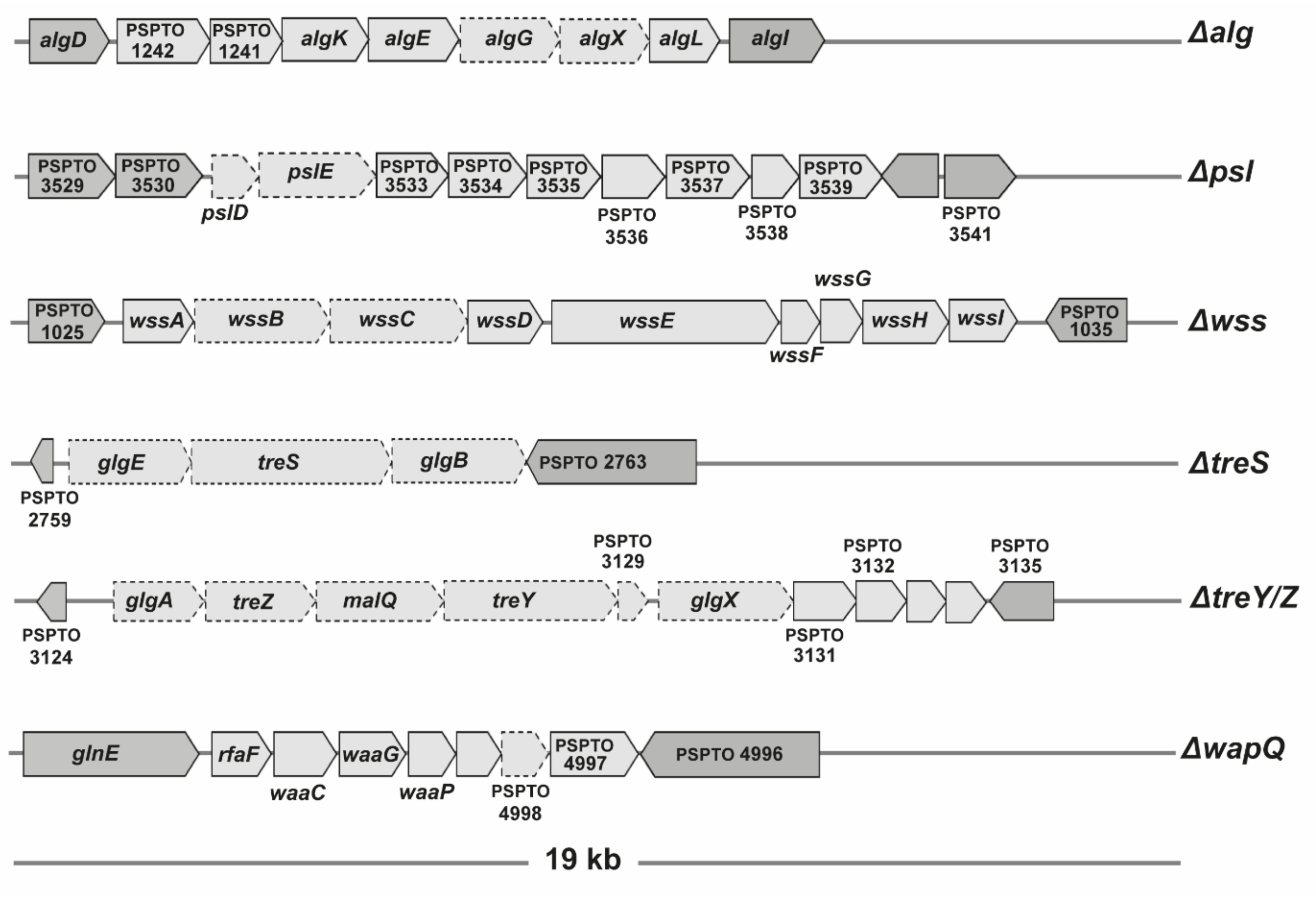

Fig. S1. Genome context and mutagenesis of EPS producing genes and a putative *wapQ gene.* Polysaccharide operons investigated in this study. Operons/gene clusters involved in production of alginate, Psl, Wss (cellulose), α-glucan and WapQ (putative LPS kinase) are shown in light grey. Upstream and downstream ORFs are shown in dark grey. The portion of the operon deleted in each case is represented with dotted lines, with the resulting mutant name indicated on the right. ORF representations are not to scale.

Figure S2 : Comparison of proteins encoded by the alginate gene clusters of *Pseudomonas syringae* pv. *tomato* DC3000 (*Pst*), *P. syringae* pv. *syringae* B728a (*Pss* B728a) and *P. aeruginosa* PAO1 (PAO1)

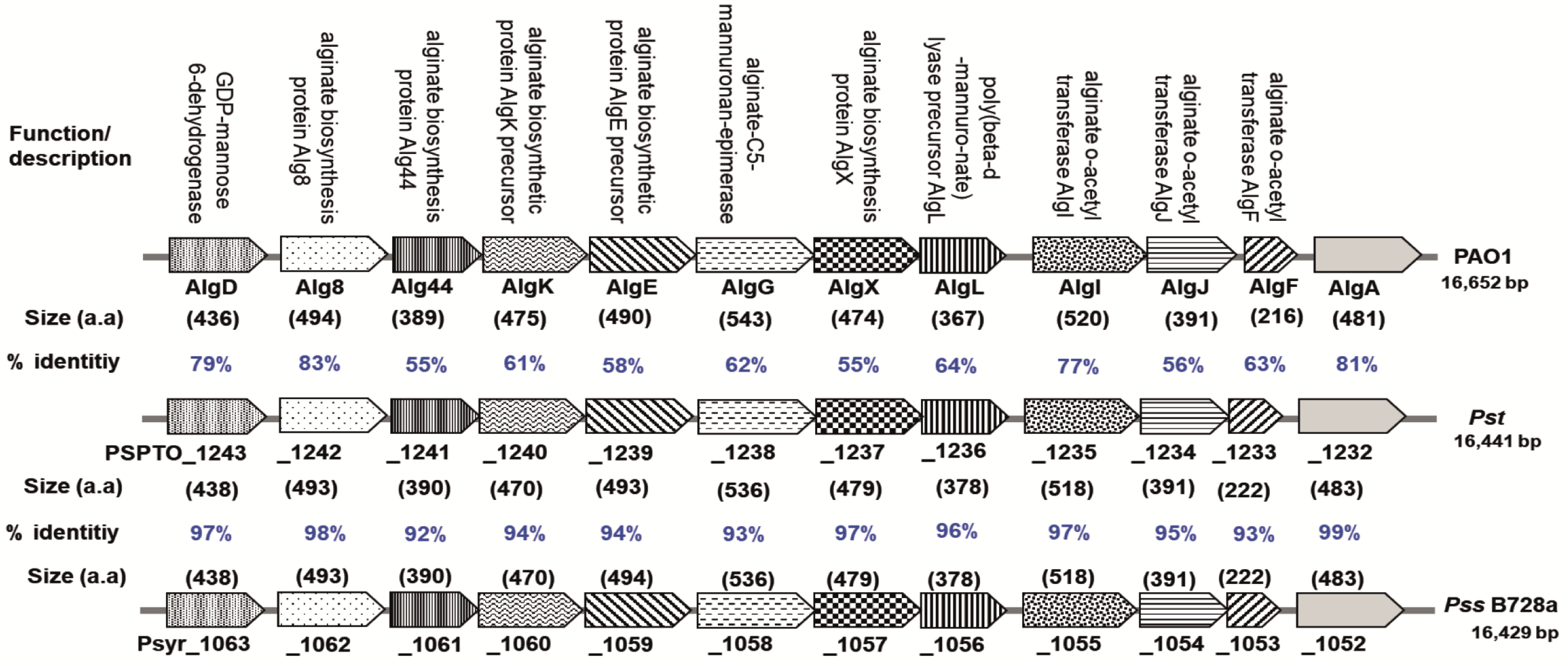

Fig. S2. Comparison of proteins encoded by the alginate gene clusters of *Pseudomonas syringae* pv. *tomato* DC3000 (*Pst*), *Pseudomonas syringae* pv. *syringae* B728a (*Pss* B728a) and *Pseudomonas aeruginosa* PAO1 (PAO1). The *PSPTO1243 - PSPTO1232* region of *Pst* encodes for alginate polysaccharide, and an orthologue region in PAO1 and *Pss* B728a were shown. The figure shows the sizes of the proteins as the number of amino acids, and the percentage of identity with that of *Pst*. Predicted functions/description have been obtained from the Pseudomonas Genome Database (https://www.pseudomonas.com/).

Figure S3 Comparison of proteins encoded by the cellulose/wss gene clusters of *Pseudomonas syringae* pv. *tomato* DC3000 (*Pst*) and *P. fluorescens* SBW25 (SBW25).

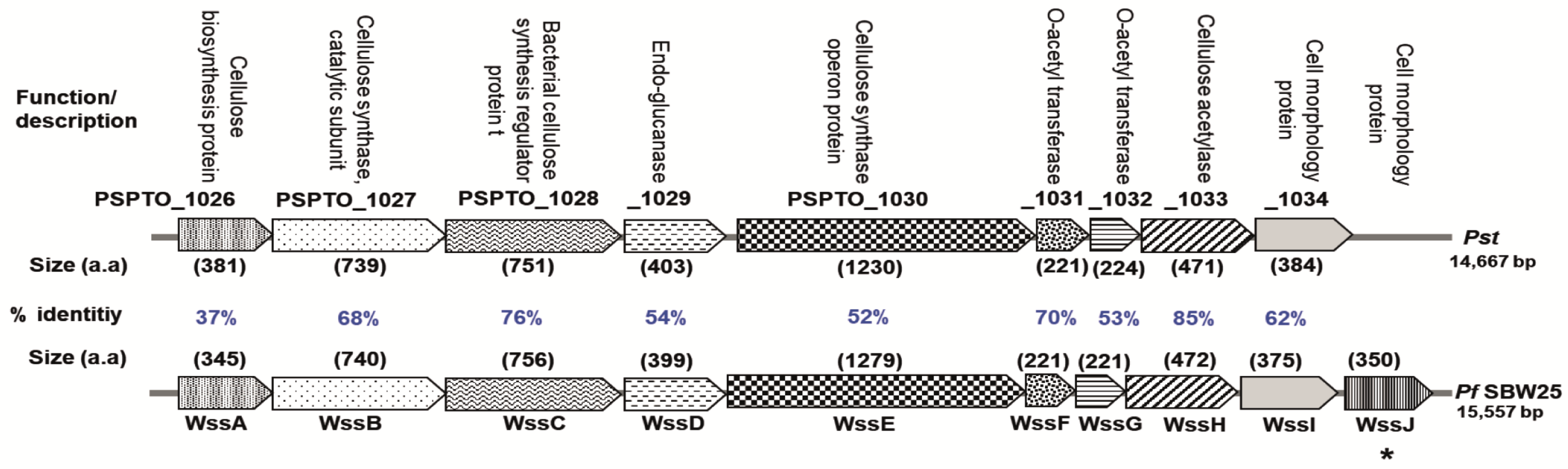

Fig. S3. Comparison of proteins encoded by the cellulose/Wss gene clusters of *Pseudomonas syringae* pv. *tomato* DC3000 (*Pst*) and *P. fluorescens* SBW25 (*Pf* SBW25). Wss operon region of *Pst* that encodes for cellulose biosynthesis pathway and an orthologue region in *Pf* SBW25 were shown. The figure shows the sizes of the proteins as the number of amino acids, and the percentage of identity with that of *Pst*. Predicted function/description have been obtained from the Pseudomonas genome Database (https://www.pseudomonas.com/). Asterisk on ORF indicates absence of orthologue in gene cluster of comparing strain.

Figure S4 : Comparison of proteins encoded by the Psl gene clusters of *Pseudomonas syringae* pv. *tomato* DC3000 (*Pst*), *P. syringae* pv. *syringae* B728a (*Pss* B728a) and *P. aeruginosa* PAO1 (PAO1)

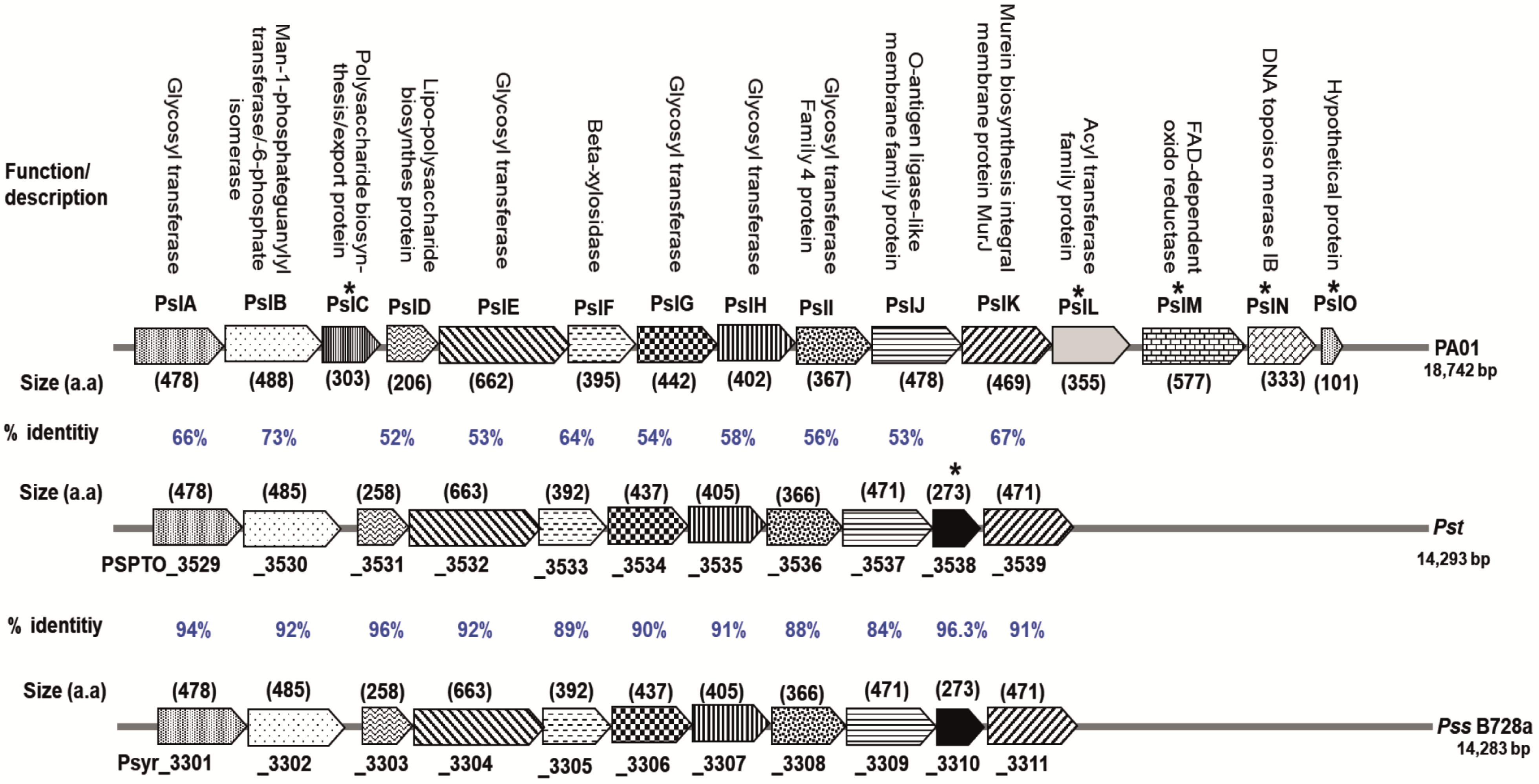

Fig. S4. Comparison of proteins encoded by the Psl gene clusters of *Pseudomonas syringae* pv. *tomato* DC3000 (*Pst*), *P. syringae* pv. *syringae* B728a (*Pss* B728a) and *P. aeruginosa* PAO1 (PAO1). The PSPTO3529 – PSPTO3539 region of *Pst* encodes for Psl polysaccharide, and an orthologue region in PAO1 and *Pss* B728a were shown. The figure shows the sizes of the proteins as the number of amino acidsand the percentage of identity with that of *Pst*. Predicted function/description have been obtained from the *Pseudomonas* Genome Database (https://www.pseudomonas.com/). Asterisk indicates absence of orthologue in gene cluster of comparing strain.

Figure S5 : Comparison of proteins encoded by the trehalose/α- glucan gene cluster-1 of *Pseudomonas syringae* pv. *tomato* DC3000 (*Pst*), *P. syringae* pv. *actinidiae* NZ-45 (*Psa*) and *P. aeruginosa* PAO1 (PAO1)

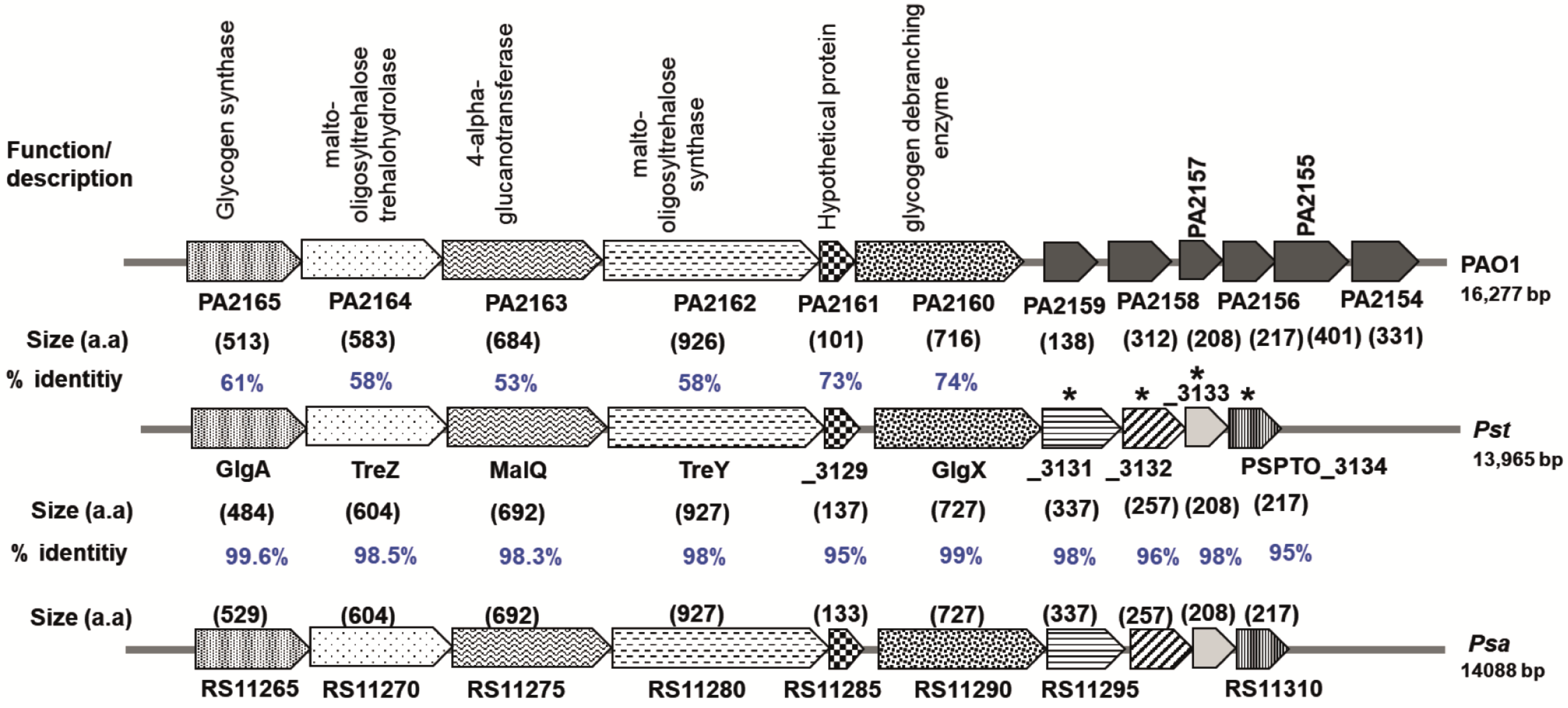

Fig. S5. Comparison of proteins encoded by the trehalose/α- glucan gene cluster-1 of *Pseudomonas syringae* pv. *tomato* DC3000 (*Pst*), *P. syringae* pv. *actinidiae* NZ-45 (*Psa*) and *P.* aeruginosa PAO1 (PAO1). The PSPTO3125 - PSPTO3134 region of *Pst* encodes for proteins involved in production of α-glucan polysaccharide, and an orthologue region in PAO1 and *Psa* were shown. The figure shows the sizes of the proteins as the number of amino acids and the percentage of identity between the two strains being compared. Predicted function/description have been obtained from the Pseudomonas Genome Database (https://www.pseudomonas.com/).

Figure S6 : Comparison of proteins encoded by the trehalose/α- glucan gene cluster-2 of *Pseudomonas syringae* pv. *tomato* DC3000 (*Pst*), *P. syringae* pv. *actinidiae* NZ-45 (*Psa*) and *P.* aeruginosa PAO1 (PAO1)

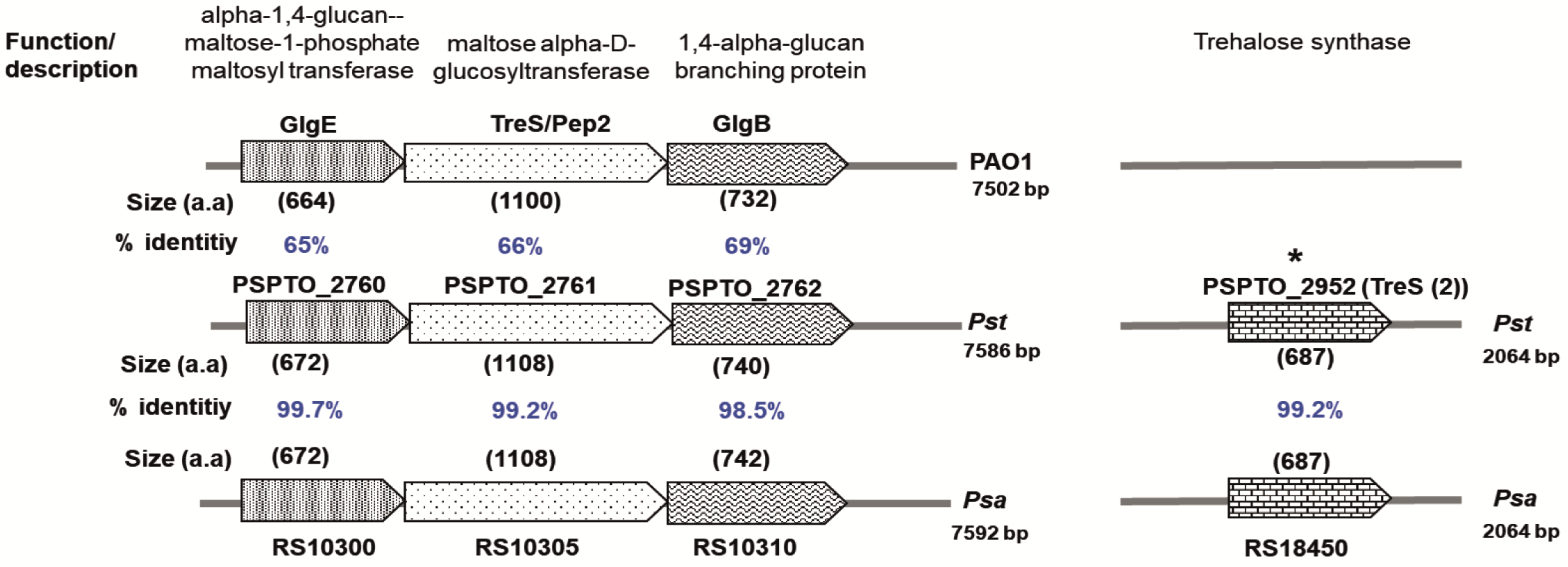

Fig. S6. Comparison of proteins encoded by the trehalose/α- glucan gene cluster-2 and a paralog of Tres (Tres(2)) of *Pseudomonas syringae* pv. *tomato* DC3000 (*Pst*), *P. syringae* pv. *actinidiae* NZ-45 (*Psa*) and *P.* aeruginosa PAO1 (PAO1). The PSPTO2760 - PSPTO2762 region of *Pst* encodes for α-glucan polysaccharide, and an orthologue region in PAO1 and *Psa* were shown. The figure shows the sizes of the proteins as the number of amino acids and the percentage of identity between the two strains being compared. Predicted function/description have been obtained from the Pseudomonas Genome Database (https://www.pseudomonas.com/). Asterisk indicates that ortholog is absent in the comparing strain.

Figure S7 : A scheme representing the pathway for the production of α-glucan in *Pst*

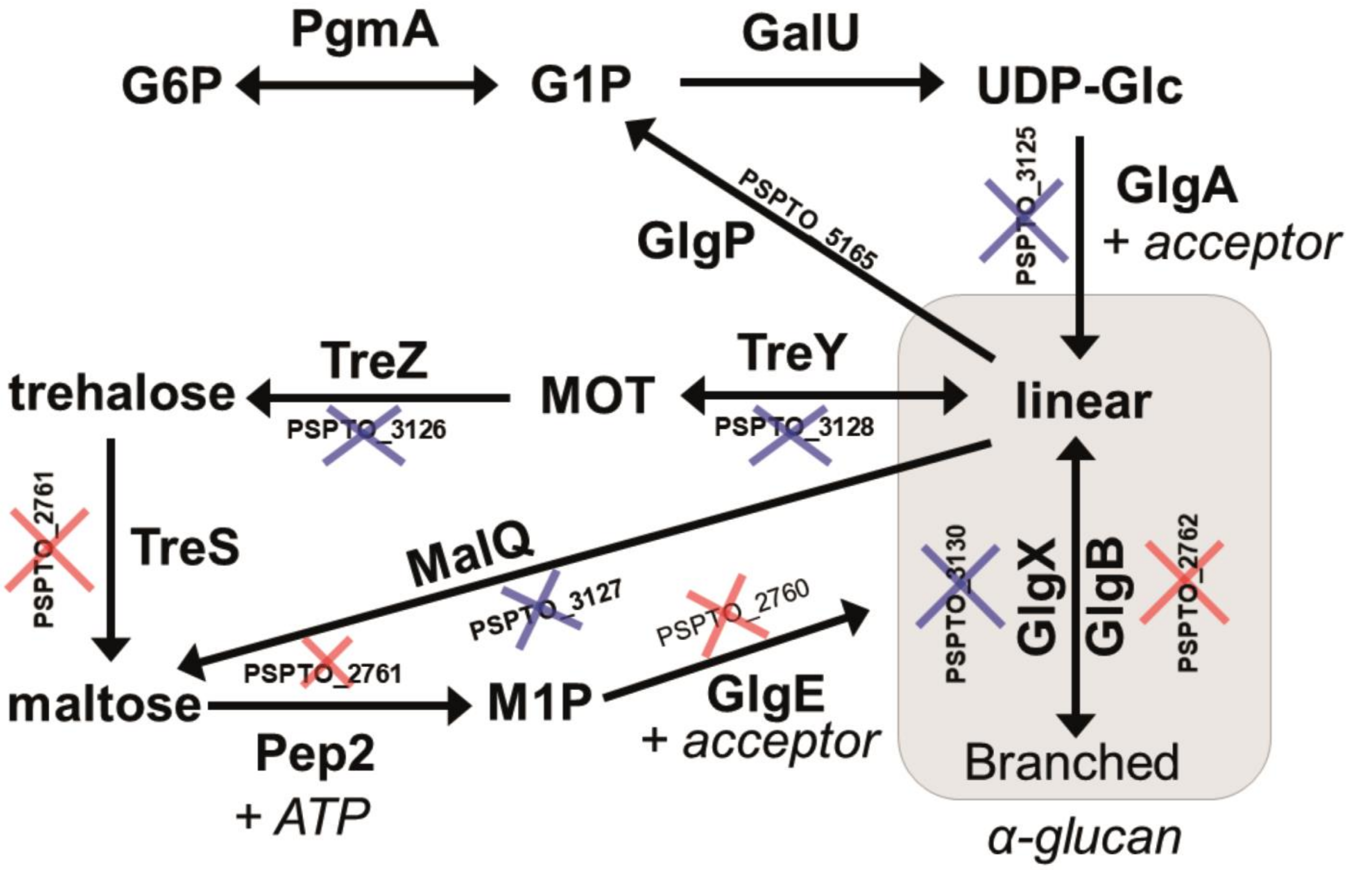

Fig S7. A scheme representing the pathway for the production of α-glucan in *Pst*. A scheme modified from Woodcock et al., 2021. Names of the corresponding gene/ORF number were presented, names correspond to respective operons shown in Fig. S1. Arrows indicate the primary direction of flux between substrates. Abbreviations are used as follows: G6P –glucose6-phosphate, G1P –glucose-1-phosphate, UDP-Glc–UDP-glucose, MOT–maltooligosyltrehalose, M1P –maltose-1-phosphate. Genes deleted in Δ*treS* mutant were shown using red cross marks and genes deleted in Δ*treY/Z* were represented using blue cross.

Figure S8: Comparison of WapQ proteins sequences encoded by different pathovars of *P. syringae* and their alignment, *P. aeruginosa* PAO1 (PAO1) and *P. fluorescens* SBW25 are the outgroup members

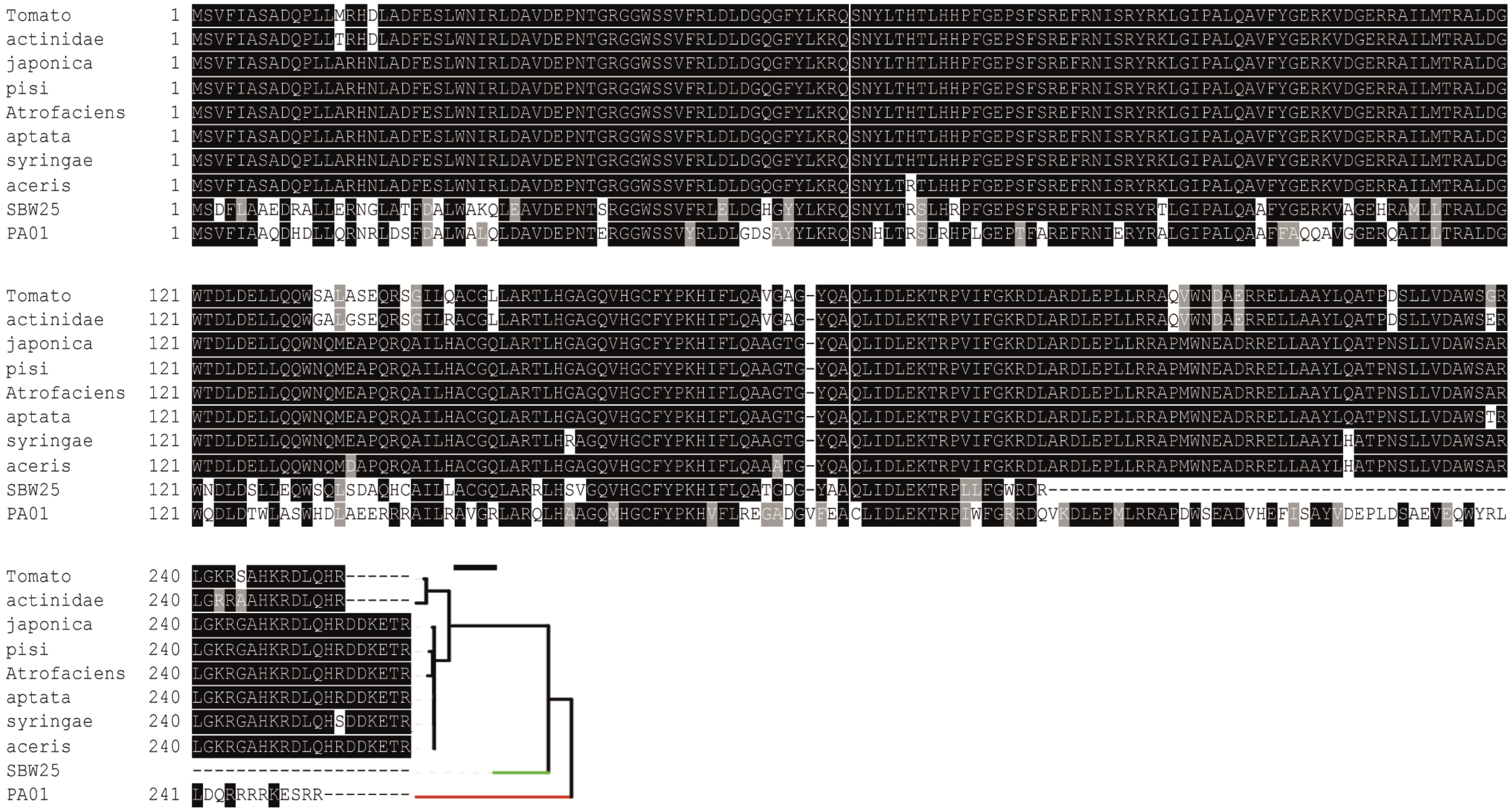

Fig. S8. Comparison of WapQ protein sequences encoded by different pathovars of *P. syringae* and their alignment, *P. aeruginosa* PAO1 (PAO1) and *P. fluorescens* SBW25 (SBW25) are the outgroup members. The protein sequence encoded by PSPTO4998 (WapQ) labelled tomato in the figure was aligned with its orthologues from other pathovars of *P. syringae* (top 8 sequences, black line, represented with pathovar name) and outgroup members PAO1 (PA5007 red line) and SBW25 (PFLU0467 green line) in the phylogenetic tree. Scale bar in the phylogenetic tree = 0.1. Orthologue sequences were obtained from the Pseudomonas Genome Database (https://www.pseudomonas.com/).

Figure S9 : Absence of WapQ results in altered LPS profile of *Pst* but no accumulation of polysaccharides in cell.

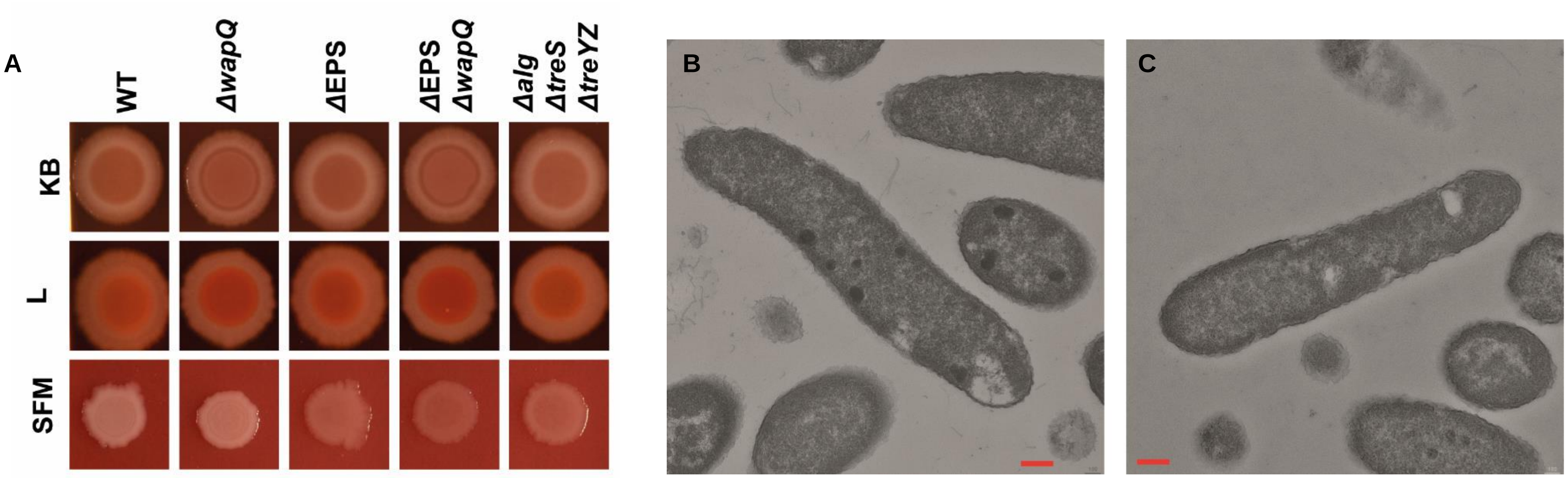

Fig S9. Absence of WapQ results in altered LPS profile of *Pst* but no accumulation of polysaccharides in cell. (A) Colony morphology of different mutant strains of *Pst* (as indicated in the figure) grown at 28 ^{o}C . (B) TEM image of sectioned WT cells. (C) TEM image of sectioned Δ*wapQ* cells. 22K X magnified TEM images. Scale bar = 200 nm.

Figure S10: Effect of low temperature on colony phenotype of different mutant strains

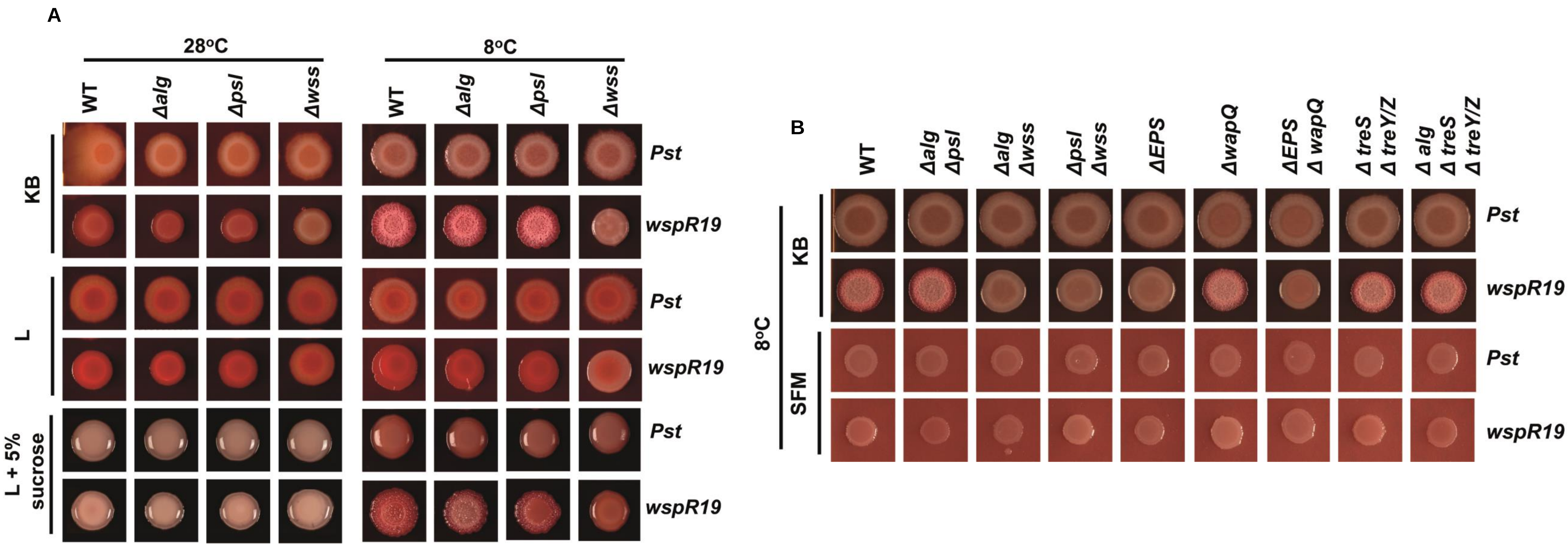

Fig S10. Effect of low temperature on colony phenotype of different mutant strains. Colony morphology of different mutant strains as indicated in the figure in *Pst* and in a strain with high cellular cdG (*wspR19*) on CR supplemented King's B medium (KB), L nutrient medium (L). L supplemented with 5% sucrose (L+5% sucrose), soy flour mannitol medium (SFM). (A) Single mutants and (B) double, triple and quadruple mutants as indicated (8 °C). Similar results were obtained in at least two independent experiments and a representative pic was shown.

Figure S11 : Disease symptoms on col-0 plants post spray infection

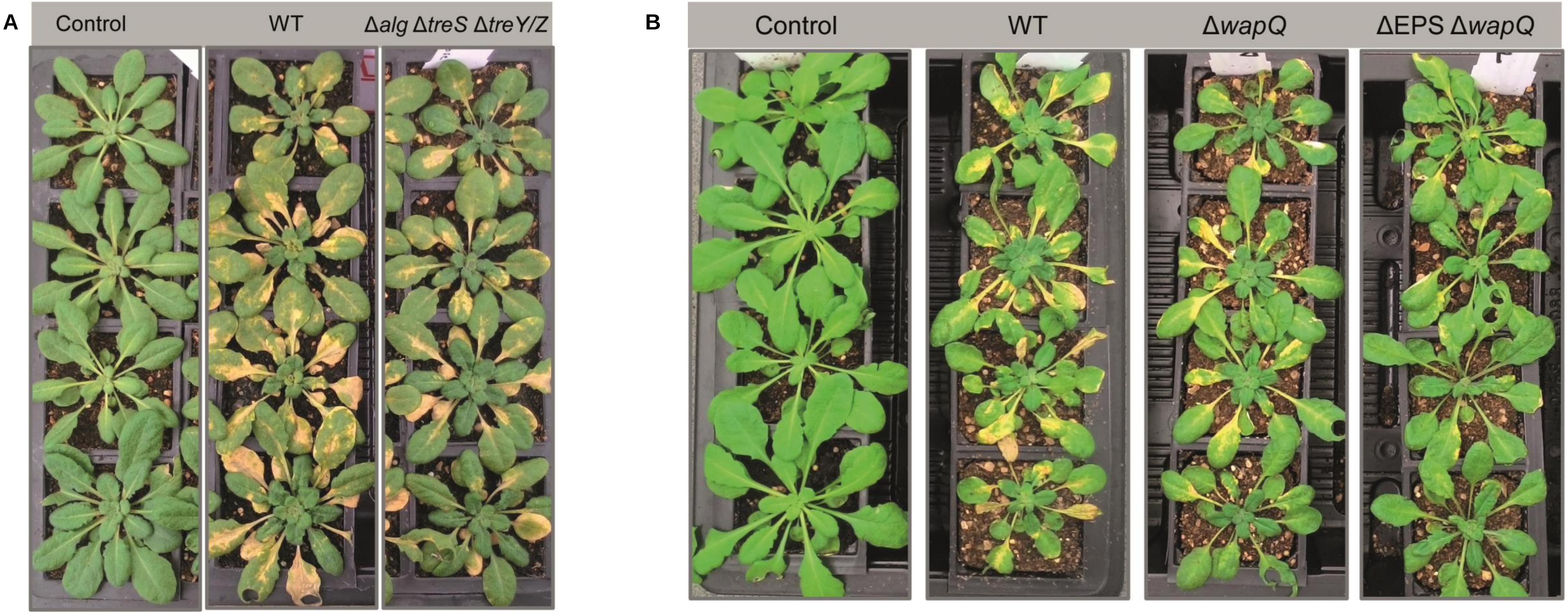

Fig S11. Disease symptoms on Col-0 plants post spray infection. (A) & (B) Plants were pictured 5 days post spray infection with different mutant strains as indicated in figure and plants were not watered post infection. Similar results were obtained for at least two independent experiments in each case and a representative image was shown.

Figure S12 : Absence of alginate and α-glucan, EPS and WapQ have no effect in infection upon infiltration

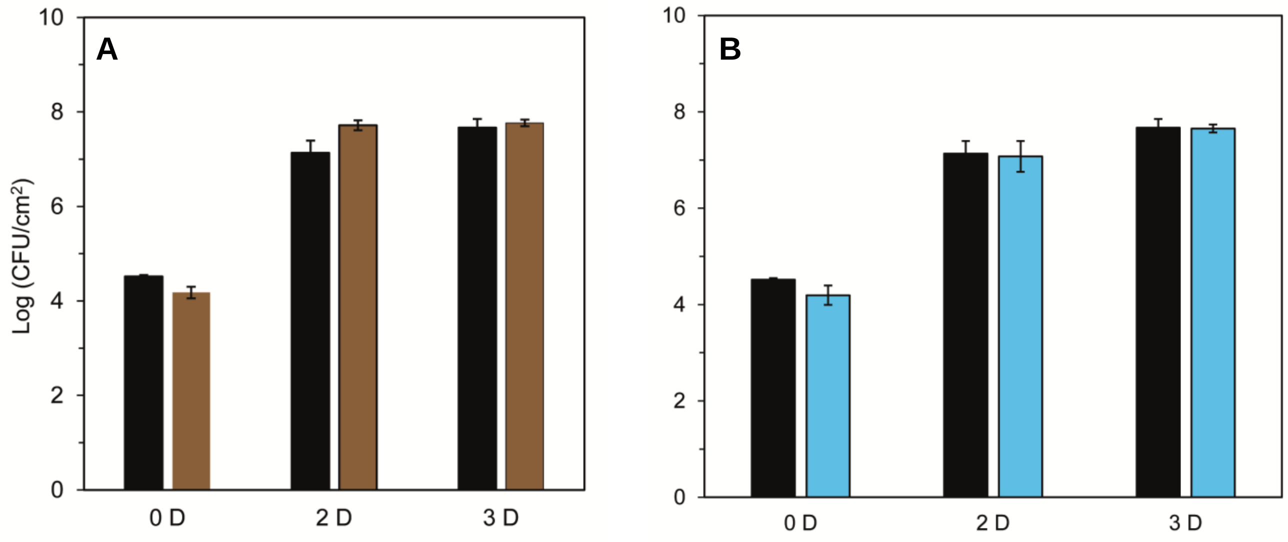

Fig S12. Absence of alginate and α-glucan, EPS and WapQ have no effect in infection upon infiltration. (A) & (B) Bacterial load following infiltration at different days post infection [D]. Black (WT), brown (Δ*alg* Δ*treS* Δ*treY/Z*) and blue (Δ*EPS* Δ*wapQ*). Similar results were obtained for at least two independent experiments in each case. Error bars represent mean ± SD from three technical replicates of a representative experiment.

Figure S13 : Absence of alginate and α-glucan has no effect on plant infection by *Pst* when plants are watered.

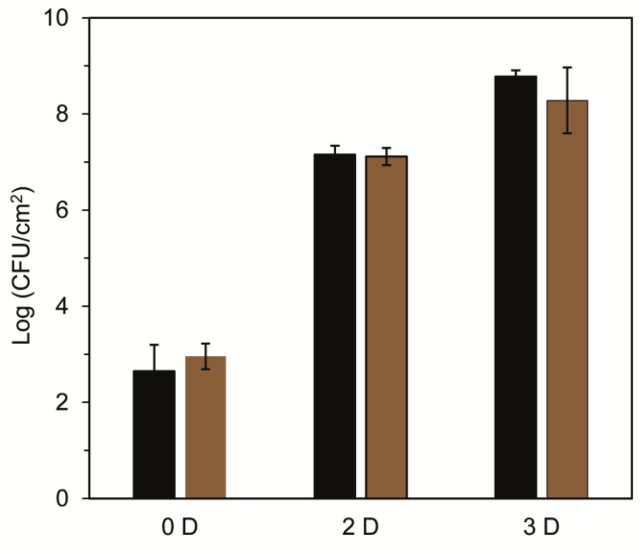

Fig S13. Absence of alginate and α-glucan has no effect on plant infection (spray) when plants are watered. (A) Bacterial load following spray infection at different days post infection [D] and plants were watered post infection. Black (WT), and brown (Δ*alg* Δ*treS* Δ*treY/Z*). Similar results were obtained for at least two independent experiments in each case. Error bars represent mean ± SD from three technical replicates of a representative experiment.

Table S1. List of primers used in this study

Target/purpose		No	Primer name	Sequence (5'-3')
Alginate mutagenesis		1	PSPTO1237/8-UPF	CGGGATCCAAGGACTACATCCAGAAC
		2	PSPTO1237/8-UPR	CGTCTAGAGCTGTTCATCTCTGGCAC
		3	PSPTO1237/8-DNF	CGTCTAGAGCTGGACTATGAGGTGAC
		4	PSPTO1237/8-DNR	CGCATATGAAGCAGAGGACATGCTGC
		5	PSPTO1237/8-Test-F	GGCTGGGCCACCGACCTGGGC
		6	PSPTO1237/8-Test-R	CAGCGGCAGATTGCTCCAGTC
Psl mutagenesis		7	PSPTO3531/2-UPF	CGGGATCCGTCGGTCTCGATAACCTG
		8	PSPTO3531/2-UPR	CGTCTAGACATCGTTCCTGAGCTCCTG
		9	PSPTO3531/2-DNF	CGTCTAGAATAGGGAGCCGTGGCTGATG
		10	PSPTO3531/2-DNR	CGGAATTCGATCACCACCATCTGCGTGG
		11	PSPTO3531/2Test-F	GTCGCAGCCAGGACGTCAAG
		12	PSPTO3531/2Test-R	CAGGCTCGATGGCCACGTTG
Wss/cellulose mutagenesis		13	PSPTO1027/8-UPF	CGGGATCCTTGATGAGCGTCGTTCTC
		14	PSPTO1027/8-UPR	CGTCTAGACGACAGGTTAGTCATGAC
		15	PSPTO1027/8-DNF	CGTCTAGAGAATAAGTCATGCCGACC
		16	PSPTO1027/8-DNR	CGCATATGAACGGTCTTGAGCAGTGC
		17	PSPTO1027/8-Test-F	GTTGGCCTATGGCTCGCTGC
		18	PSPTO1027/8-Test-R	GGCAGGTTGACCACCAGCC
wapQ (*PSPTO4998*) mutagenesis		19	PSPTO4998-UPF	AGTCGACCTGCAGGCATGCAAAGCGCTTCGCCAAATGG
		20	PSPTO4998-UPR	GTTGAAGGTCAATAAAAACGCTCATTCACGGC
		21	PSPTO4998-DNF	CGTTTTTATTGACCTTCAACACCGATGATTTC
		22	PSPTO4998-DNR	GACCAATTGACTACCTAGGATCCAGGTGCAGATCTTCC
		23	PSPTO4998-Test-F	CCGACAAGTTGTATCAGCGTAAAGTGCG
		24	PSPTO4998-Test-R	CAGGCTGCAATCGCGCACGGTCTTGC
α-glucan Δ*treS* mutagenesis	*glgE* (*PSPTO2760*)	25	PSPTO2760-UPF	CGCGAAGCTTGCACATTCATTGACCTGG
		26	PSPTO2760-UPR	CGCGTCTAGACTGTTCATTCATCGAGC
		27	PSPTO2760-DNF	CGCGTCTAGAGGAATCTGGCGTATC
		28	PSPTO2760-DNR	CGCGGGATCCGTTGGATGTCTCGGTGTC
		29	PSPTO2760-Test-F	TCAGACAACGCATCGTATGC
		30	PSPTO2760-Test-R	AACCGGTGCCAGAAATACTG
	treS/*pep2* (*PSPTO2761*)	31	PSPTO2761-UPF	CGGGATCCGACATCAATCCGCGCTTC
		32	PSPTO2761-UPR	CGTCTAGACGCGGCATCGGGCTTC
		33	PSPTO2761-DNF	CGTCTAGAGGGTTGCTGCAACCGTC
		34	PSPTO2761-DNR	CGGGTACCAGACGCGGAACAAAGATC
		35	PSPTO2761-Test-F	GAATTTCTTCGTCAACAC
		36	PSPTO2761-Test-R	CTCGTATTTGTAGACCTC
	glgB (*PSPTO2762*)	37	PSPTO2762-UPF	CGCGAAGCTTAGTTGGTCAGCGACCTG
		38	PSPTO2762-UPR	CGCGTCTAGACGCATTCATATCGGTTC
		39	PSPTO2762-DNF	CGCGTCTAGAGCTTCTCTGACACTGAATC
		40	PSPTO2762-DNR	CGCGGGATCCAAGGCCATGCGCTAAG
		41	PSPTO2762-Test-F	TCTGGACAAGGATGATCAG

		42	PSPTO2762-Test-R	TCGATGCGCTGGTGATGAG
α-glucan ΔtreY/Z mutagenesis	*glgA* (*PSPTO 3125*)	43	PSPTO3125-UPF	CGCGGGATCCGATGAGGCTCGTCAC
		44	PSPTO3125-UPR	CGCGTCTAGAGTTGACCTGAGACATG
		45	PSPTO3125-DNF	CGCGTCTAGAGTGAGCATGACCAGCAA
		46	PSPTO3125-DNR	CGCGAAGCTTTGTTTGAGCTGTTCG
		47	PSPTO3125-Test-F	CGGAGATTGAACATCAGG
		48	PSPTO3125-Test-R	AGAATCACCATCAGGCCAACG
	treZ (*PSPTO 3126*)	49	PSPTO3126-UPF	CGCGGGATCCACATCGTCAACAACGG
		50	PSPTO3126-UPR	CGCGTCTAGAATCGTCTGTACGCAGG
		51	PSPTO3126-DNF	CGCGTCTAGAGCAAGTGATGTTCTGGAG
		52	PSPTO3126-DNR	CGCGAAGCTTGCAGGCTGTAAAGCTG
		53	PSPTO3126-Test-F	CACCTGATCTGTCACTTCG
		54	PSPTO3126-Test-R	AGCATTTCCAGTGCCTCC
	malQ (*PSPTO 3127*)	55	PSPTO3127-UPF	CGCGGGATCCTTCATGGGCGATGAATG
		56	PSPTO3127-UPR	CGCGTCTAGAAAGCCTCTCCAATTGC
		57	PSPTO3127-DNF	CGCGTCTAGACGACTGGAATTGCTGTC
		58	PSPTO3127-DNR	CGCGAAGCTTCTGGTAGTGCTCGATG
		59	PSPTO3127-Test-F	ACTGCGCTGTTGTTGCTCTC
		60	PSPTO3127-Test-R	GTTCCACTGTCTGCAATAAC
	treY (*PSPTO 3128*)	61	PSPTO3128-UPF	CGCGGGATCCATTGGTCCGATCAGGCAC
		62	PSPTO3128-UPR	CGCGTCTAGAGTCATCAAGGGTGAAG
		63	PSPTO3128-DNF	CGCGTCTAGAATACCGCTCAGTGCAGC
		64	PSPTO3128-DNR	CGCGAAGCTTCGTCTCTGGATCAGAG
		65	PSPTO3128-Test-F	CTGTTGTTCGAACAGAAC
		66	PSPTO3128-Test-R	ATTCATGGTGCCTTCCTGACG
	glgX (*PSPTO 3128*)	67	PSPTO3130-UPF	CGCGGGATCCGTCATGATGAGCGTCAC
		68	PSPTO3130-UPR	CGCGTCTAGAAGAACTGTCGTGCGAAG
		69	PSPTO3130-DNF	CGCGTCTAGAGAAGTCAAGCGTGAAG
		70	PSPTO3130-DNR	CGCGAAGCTTTCGGCATTCTCGAACAC
		71	PSPTO3130-Test-F	CCTATCAGATCTGGGAATC
		72	PSPTO3130-Test-R	TCTTCACGTTTGCCGATGTG
Reporter		73	PSPTO3125-UPFP	CGCGGGATCCGATGAGGCTCGTCAC
		74	PSPTO3125-UPRP2	CGCGAAGCTTGCCTGTTGACCTGAGACATG
Transposon (Tn-seq)		75	Arb-PCR	CGCAAACCAACCCTTGGCAG
		76	Arb1b	GGCCAGCGAGCTAACGAGACNNNNGATAT
		77	Arb1	GGCCAGCGAGCTAACGAGAC
		78	Almar3-seq	ACATATCCATCGCGTCCGCC
Real time PCR (qRT)		79	16S-RT-F	ACGGGTACTTGTACCTGGTG
		80	16S-RT-R	CGTTTCCGAGCGTTATCCC
		81	gyrA-RT-F	GAAGGGCAGAAGCTGATTT
		82	gyrA-RT-R	ACTCAGGGAACTCGGAAA
		83	algX-RT-F	TGGACCAGGAGACCATCTATC
		84	algX-RT-R	TGACCAGCACTTCGTTGTTGC
		85	pslD-RT-F	GGCCACCAACCTCAATCAAGC
		86	pslD-RT-R	CCAGAGAAACTTGCTGTAGTCG
		87	wssB-RT-F	GTGGTCGAGGAGAAGTTCTTTG
		88	wssB-RT-R	GTTGTTCCAGCCCAGTTGATAC
		89	glgA-RT-F	AAATCAACGCCGATTACGTC
		90	glgA-RT-R	CAGGCCTTTCTGGTAAACGA
		91	*wapQ*-RT-F	ACGACCTGGCTGATTTTGAATCG
		92	*wapQ*-RT-R	GTCCAGATCAAGGCGAAACACAC
